# Boron-doped sodium layered oxide for reversible oxygen redox reaction in Na-ion battery cathodes

**DOI:** 10.1038/s41467-021-25610-7

**Published:** 2021-09-06

**Authors:** Yu-Jie Guo, Peng-Fei Wang, Yu-Bin Niu, Xu-Dong Zhang, Qinghao Li, Xiqian Yu, Min Fan, Wan-Ping Chen, Yang Yu, Xiangfeng Liu, Qinghai Meng, Sen Xin, Ya-Xia Yin, Yu-Guo Guo

**Affiliations:** 1 CAS Key Laboratory of Molecular Nanostructure and Nanotechnology, CAS Research/Education Center for Excellence in Molecular Sciences, Beijing National Laboratory for Molecular Sciences, Institute of Chemistry, Chinese Academy of Sciences (CAS), Beijing, People’s Republic of China; 2 University of Chinese Academy of Sciences, Beijing, People’s Republic of China; 3 Beijing National Laboratory for Condensed Matter Physics, Institute of Physics, CAS, Beijing, People’s Republic of China

**Keywords:** Batteries, Materials chemistry, Batteries, Batteries

## Abstract

Na-ion cathode materials operating at high voltage with a stable cycling behavior are needed to develop future high-energy Na-ion cells. However, the irreversible oxygen redox reaction at the high-voltage region in sodium layered cathode materials generates structural instability and poor capacity retention upon cycling. Here, we report a doping strategy by incorporating light-weight boron into the cathode active material lattice to decrease the irreversible oxygen oxidation at high voltages (i.e., >4.0 V vs. Na^+^/Na). The presence of covalent B–O bonds and the negative charges of the oxygen atoms ensures a robust ligand framework for the NaLi_1/9_Ni_2/9_Fe_2/9_Mn_4/9_O_2_ cathode material while mitigating the excessive oxidation of oxygen for charge compensation and avoiding irreversible structural changes during cell operation. The B-doped cathode material promotes reversible transition metal redox reaction enabling a room-temperature capacity of 160.5 mAh g^−1^ at 25 mA g^−1^ and capacity retention of 82.8% after 200 cycles at 250 mA g^−1^. A 71.28 mAh single-coated lab-scale Na-ion pouch cell comprising a pre-sodiated hard carbon-based anode and B-doped cathode material is also reported as proof of concept.

## Abstract (web summary)

The irreversible oxygen redox reaction during charging to the high-voltage region causes cathode structural degradation and Na-ion cell capacity fading. Here, the authors report a B-doped cathode active material to mitigate the irreversible oxygen oxidation and increase the cell capacity.

## Introduction

Na-ion batteries (NIBs) have been recognized as sustainable solutions to alleviate the resource anxiety of Li-based electrochemical energy storage mainly from sufficient and low-cost sodium raw materials^1,2^. The growing proliferation of robust layered cathode and carbon anode materials has been intensively reported in the last few years. However, the practical implementation of NIBs faced the issue of lower specific energy due to the relatively heavier and less-reducing potential of Na compared with Li. Specifically, the Cost-Per-kWh of NIBs should be reduced to increase their market competitiveness against Li-ion batteries (LIBs). To achieve the goal, a feasible strategy is to improve the specific energy and energy density at the cell level. As for the cathode active material, it is required to be reversibly operated at a high voltage and with a large Na^+^ extraction. Anion redox reaction that occurs during high voltage desodiation of layered oxide cathodes offers an avenue to realizing high-energy-density NIBs as it provides an additional capacity by storing charges on both transition metal cations and oxygen anion.

The sodium storage mechanism has been intensively proposed on the key points to motivate the anionic redox that reported in Li-rich compounds from the last two decades^3–8^. Ceder and co-workers proposed the origin of anionic redox in Li-rich and cation-disordered oxides^5^: the formation of Li–O–Li interactions function as the impetus to trigger oxygen redox due to the weak overlap between O 2*p* and Li 2*s* orbitals, thus favoring the O *2p* band approximate to the Fermi level and facilitating the oxygen redox reaction. Such a similar situation has been observed in Na-rich and Na-deficient layered oxides^9–14^. These formed Na–O–Na, Na–O–Li, Na–O–Mg interactions are generally considered to effectively trigger oxygen redox, due to the formation of nearly non-bonding state oxygen^10,15,16^. The as-triggered anionic (oxygen) redox processes, combined with cationic redox reaction, enable the staggering capacity increases of layered compounds. However, layered oxides involving oxygen redox reaction commonly suffer from the irreversible oxygen loss from the lattice upon charged to high voltage (>4.0 V), which eventually results in structural degradation and poor electrochemical performance^17,18^. In addition, the irreversible oxygen release at the surface of layered compounds accelerates the decomposition of electrolyte, which is gradually accumulated on the surface of cathodes, thereby yielding the thick and resistive cathode electrolyte interphase (CEI), and thus resulting in sluggish Na^+^ transfer kinetics^19^. Therefore, suppressing irreversible oxygen release upon deep desodiaton is crucial for constructing high-capacity cathode materials and actuating the development of NIBs.

Typical improvement strategies by lowering the operation voltage, or reinforcing the metal-oxygen bond via coating and doping are used to suppress oxygen release. While lowering the operation voltage intends to avoid the occurrence of anion redox, it also accounts for dramatically reduced specific energy and energy density of cathode as both Na-storage capacity and voltage are concurrently decreased. This incurs a dilemma in the research of NIBs: the tradeoff between high-voltage electrochemical stability and specific energy and energy density of cathode material. Inert surface coating of metal oxide (Al_2_O_3_, MgO)^20,21^ or metal phosphate (NaPO_3_, β-NaCaPO_4_)^17,22^ were utilized to stabilize the crystal structure through suppressing surface irreversible phase transformation from irreversible oxygen redox reaction. Alternatively, the doping of inactive cations (Ti^4+^, Se^6+^, Nb^5+^)^23–25^ is used to increase the oxygen binding energy effectively^26^, alleviating the irreversible oxygen redox reaction and promoting the stability of crystal structure^27–29^. However, it is still challenging to achieve a highly homogenous coating layer on the surface of cathode particles. Doping of heavy inactive metal ions inevitably leads to the reduced specific capacity of cathode^30–32^, for instance, it will cause a capacity decrement of ca. 7.3% by introducing 5 atom.% of Zn^2+^ dopant into the lattice of Na_0.833_[Li_0.25_Mn_0.75_]O_2_ compound, and 16.0% by 10 atom.% of Ti^4+^ dopant in the Na_0.72_Li_0.24_Mn_0.76_O_2_ compound according to the previous works (Supplementary Table 1)^13,33^.

With this perspective, doping light-weight element with strong oxygen bond might be a promising strategy to suppress reversible oxygen redox and maintain a minor capacity decrease synchronously. Several light-weight elements have been used as dopants to promote the electrochemical performance of layered oxides which solely undergo the cationic redox even being charged to a very high voltage of 4.6 V vs. Na^+^/Na. For instance, Li has been introduced into layered oxide Na_0.9_Ni_0.3_Mn_0.4_Fe_0.3_O_2_ to mitigate the Ni^3+^ Jahn–Teller distortion and prevent the loss of active transition-metal ions^34^. Boron has also been studied as a cathode dopant for Li-ion and Na-ion batteries. In the previous work by Vaalma et al., they reported that B-doping helps to stabilize the cationic redox of P2-type Na_2/3_MnO_2_ cathode material, but with complex phase transition and fast capacity degradation at high voltage^35^. It should be noted that the boron owns the highest binding energy with oxygen among the previously reported elemental dopants (809 kJ mol^−1^ for B–O, ~666.5 kJ mol^−1^ for Ti–O, ≤250 kJ mol^−1^ for Zn–O bond)^36^. The high binding energy of B–O bonds makes it capable of suppressing the loss of lattice oxygen. Li et al. reported B-doping could tune the electronic structure through the lowered O 2*p* band top, thus enhancing the stability of oxygen in Li-rich layered cathodes^37,38^.

In this work, we report a doping strategy by incorporating light-weight boron into O3-NaLi_1/9_Ni_2/9_Fe_2/9_Mn_4/9_O_2_ to suppress the irreversible oxygen release as charging to >4.0 V. We reveal, from density functional calculations, that the light-weight boron dopant forms a strong-covalent B–O bond with adjacent O atoms, making O atom electronically more negative to resist excessive oxidation during high-voltage charge compensation via anion redox. The combined analysis of operando X-ray diffraction experiments, X-ray absorption spectroscopy, and differential electrochemical mass spectrometry experimentally demonstrates that the irreversible oxygen release from the lattice at the deep desodiated state is effectively suppressed, accompanying with good structural stability. Interestingly, the light-weight B-doping triggers a more cationic redox reaction to donate an extra capacity, resulting in about a 10.1% increment in capacity compared with the control material. The B-doped O3-type NaLi_1/9_Ni_2/9_Fe_2/9_Mn_4/9_B_1/50_O_2_ (NLNFMB) exhibits a higher reversible capacity of 160.5 mA h g^−1^ at 0.1C (25 mA g^−1^) and cycling stability with 82.8% after 200 cycles at 1C (250 mA g^−1^) in a half cell, compared with that of the undoped pristine NLNFM. The resultant hard carbon||NLNFMB full cell delivers a specific energy of up to 224 Wh kg^−1^ based on the total mass of cathode and anode active materials. This research demonstrates the prospect of light-weight boron doping for NIBs technology that could suppress irreversible oxygen release and increase the capacity synchronously. Much more stable and high-capacity layered oxides with oxygen redox could be further designed through lightweight element doping strategy.

## Results

### Theoretical calculation of the boron-doped oxide compounds

**Fig. 1:**
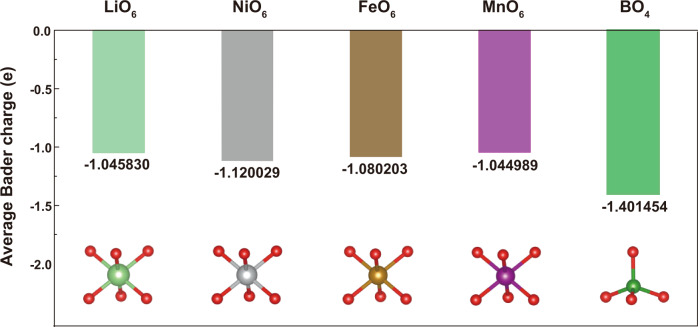
Bader charge analysis. Average Bader charge of the O atoms in MO_6_ octahedron (M = Ni/Fe/Mn/Li) and in BO_4_ tetrahedron.

**Fig. 2:**
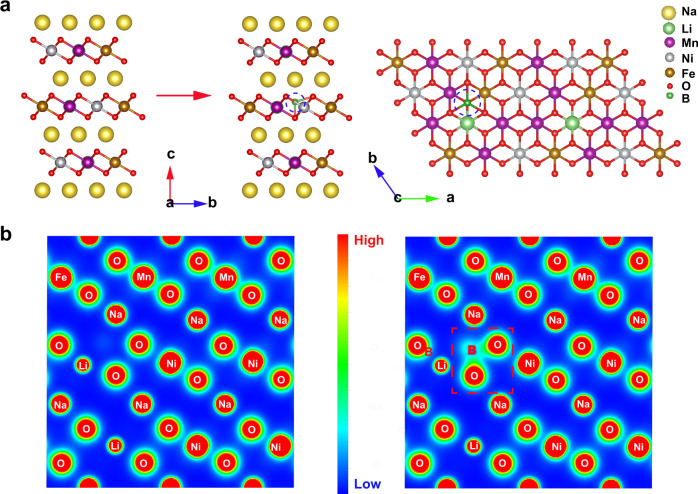
Structure and charge density analysis. Schematic structures of NLNFM and NLNFMB (**a**). Contour maps of charge density on corresponding planes in NLNFM and NLNFMB (**b**).

O3-NaLi_1/9_Ni_2/9_Fe_2/9_Mn_4/9_O_2_ (NLNFM) that is expected to have anion redox with the formation of Na–O–Li interaction, was targeted for boron doping strategy. The TM arrangement in NLNFM is displayed in Supplementary Fig. 1 and Table 2. To figure out the detailed position of boron doping in layered NLNFM, the energy of two configurations with tetrahedral and trigonal boron doping sites were calculated, respectively^37,39^. The lowest energy is achieved for the B surrounded by Li–Fe–Mn in tetrahedral interstitial sites (Supplementary Table 3). Bader charge analysis was further employed to disclose the influence of B-doping on the NLNFM structure (Fig. 1, the detailed data are summarized in Supplementary Table 4). One can clearly see that the average charge around the O atoms between the BO_4_ octahedron in the B-doped NLNFMB is more negative than those between the LiO_6_, NiO_6_, FeO_6_, MnO_6_ octahedron in NLNFM by an average extra charge of ca. −0.3 e. This demonstrates that each B–O bond introduces a more negative valence charge for O atom, which could ensure the robust O ligand framework, thus resisting excessive oxidation during high-voltage charge. The calculated more negative charge around the O atoms between the BO_4_ octahedron in the B-doped NLNFMB is consistent with the previous report that 0.02 boron per formula for Li-rich oxides can lower the top of the O 2*p* band, thus enhancing oxygen stability^37^. In addition, we calculated the O vacancy formation energy for the B-doped and undoped materials for comparison to better understand the effect of doping on oxygen release (Supplementary Table 5)^40^. O vacancy formation energy in NLNMFMB increases after B-doping. This indicates that the oxygen is difficult to escape from the lattice in B-doped material. Furthermore, charge density analysis shows that the NLNFMB possesses a stronger B–O covalent bond with introducing extra electrons for the O atom (Fig. 2), in good agreement with the result of Bader charge analysis. The light-weight B-doping is therefore prone to strengthening the oxygen ligand framework from extra negative charge for O atom, thus favoring to reduce the loss of oxygen from the lattices and improve the structural stability.

### Synthesis and structural analysis of materials

**Fig. 3:**
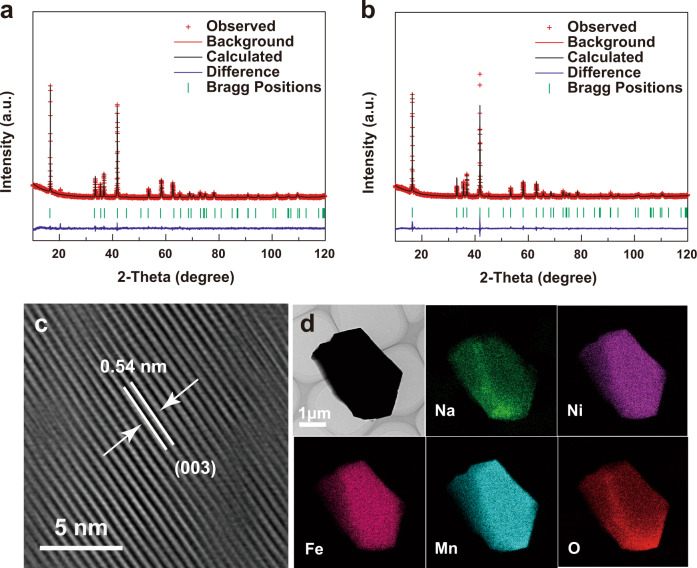
Physicochemical measurements and analyses of the cathode active material powders. XRD and Rietveld refinement results of NLNFM (**a**) and NLNFMB (**b**), respectively. HRTEM images of (003) crystal planes (**c**) and EDS maps of NLNFMB (**d**).

The NLNFM and NLNFMB samples were synthesized by a simple solid-state reaction. Inductively coupled plasma atomic emission spectroscopy (ICP-AES) was performed to confirm the stoichiometric ratio in NLNFMB. The atom ratio of Na:Li:Ni:Fe:Mn:B is 1.093:0.115:0.222:0.212:0.434:0.02 in NLNFMB, close to its chemical stoichiometric ratio. Scanning electron microscopy (SEM) image of NLNFMB shows the well-defined crystallites with ca. 13 μm in diameter (Supplementary Fig. 2a), which is larger than that of NLNFM (Supplementary Fig. 2b). All the diffraction peaks of two samples are indexed to a rhombohedral symmetry lattice with the space group R$\bar{3}$m (as shown by XRD patterns and corresponding Rietveld refinement in Fig. 3a, b), indicating an O3 phase material that sodium ions occupy octahedral sites and oxygen columns have an ABCABC-stacking mode (Supplementary Tables 6 and 7). The strong binding energy between B and O could contract the TM slabs, leading to the decreased lattice parameter *a* after B-doping. The comparison of the two samples shows that the lattice parameters *c* is increased after the B substitution, which is ascribed to the entry of extra coordinated B species with oxygen in transition metal layers. The increased electron density on oxygen has been confirmed from Bader charge calculation. The larger negative charges on oxygen, in turn, enlarge the interspacing of TMO_2_ slabs from 0.333 to 0.347 nm (Supplementary Table 8) due to the stronger electrostatic repulsion, resulting in the good Na^+^ diffusion kinetics. The energy barriers for Na-ion diffusion in layered oxide with and without B-doping were investigated in Supplementary Fig. 3. For the pristine NLNFM, the Na^+^ diffusion energy barrier is calculated to be 0.45 eV as hopping from one octahedral lattice site to its nearest vacant octahedral site by passing through a tetrahedral site. After B-doping, this diffusion barrier is lowered to 0.37 eV. This directly demonstrates that B interstitial dopant can decrease the diffusion barrier and hence facilitate the fast Na^+^ diffusion. High-resolution transmission electron microscopy (HRTEM) observation shows that the NLNFMB samples are highly crystalline (Fig. 3c), with an interfringe distance of 0.54 nm corresponding to the (003) planes in rhombohedral symmetry. Energy-dispersive spectroscopy (EDS) mapping images demonstrate that Na, Ni, Fe, Mn, and O elements are uniformly distributed in the NLNFMB particle (Fig. 3d). To check the existence of B in NLNFMB, X-ray photoelectron spectroscopy (XPS) was carried out. The signal peak of 192 eV in B region of NLNFMB is clearly visible even Ar^+^ etching 10 minutes (Supplementary Fig. 4a), demonstrating the B-doping into NLNFMB, whereas no signal peak of B 1*s* is observed for NLNFM (Supplementary Fig. 4b). ToF-SIMS depth analysis shows that the BO_2_^−^ fragment without enriching on the surface of NLNFMB compound (Supplementary Fig. 5). All above, B is uniformly distributed throughout the particles.

### Electrochemical performance

**Fig. 4:**
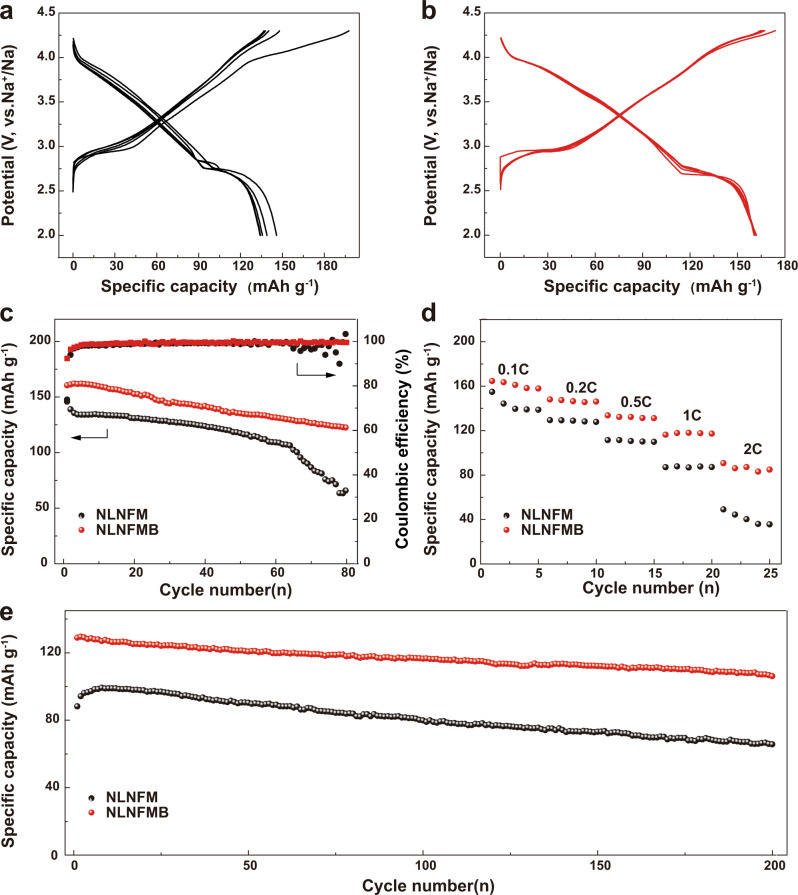
Na-ion electrochemical energy storage performances in half-cell configuration at 25 °C. Galvanostatic charge/discharge voltage profiles of NLNFM (**a**) and NLNFMB (**b**) at 25 mA g^−1^ between 2.0 and 4.3 V. Cycling performance at 25 mA g^−1^ (**c**), rate capability at various charge-discharge rates (**d**), and cycling performance at 250 mA g^−1^ (1C = 250 mA g^−1^) (**e**).

**Fig. 5:**
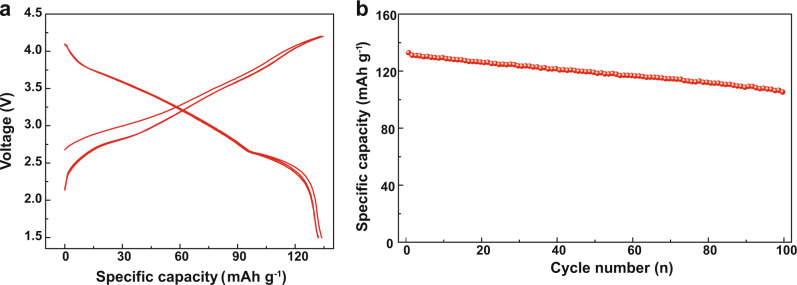
Na-ion electrochemical energy storage performances in full-cell configuration. Galvanostatic charge/discharge voltage profiles between 1.5 and 4.2 V (**a**) and cycling performance at 250 mA g^−1^ for hard carbon||NLNFMB full cell (**b**).

In order to verify the function of B substitution, the electrochemical performances of NLNFM and NLNFMB cathodes were examined in 2032 coin cells using a sodium metal negative electrode between 2.0 and 4.3 V. Figure 4a, b shows the initial five galvanostatic charge/discharge (GCD) curves of NLNFM and NLNFMB cathodes at a specific current of 25 mA g^−1^. In the first cycle, the NLNFM cathode delivers a large charge capacity of 197.5 mA h g^−1^ with a long voltage plateau above 4.1 V, which is equivalent to the removal of ~0.78 Na^+^ per formula unit. The discharge capacity is 145.8 mA h g^−1^, giving rise to a low Coulombic efficiency of 73.8% in the first cycle. From the second cycle, the plateau above 4.1 V disappears, and the Coulombic efficiency increases to 94.5%. In marked contrast to NLNFM, the NLNFMB cathode shows a charge capacity of 173.9 mA h g^−1^ (equivalent to the removal of ~0.69 Na^+^ per formula unit) and a reversible discharge capacity of 160.5 mA h g^−1^ with a higher Coulombic efficiency of 92.3%. In particular, the NLNFMB cathode exhibits a good match from the first to the fifth cycle, reflecting the good reversibility. Considering the discharge capacity (160.5 mA h g^−1^) and the average discharge voltage (3.26 V), a specific energy of 521 Wh kg^−1^ based on the mass of cathode material can be calculated for Na||NLNFMB coin cell. Note that the NLNFMB cathode exhibits the smooth sloping charge curve without a distinct plateau above 4.1 V in the first and subsequent cycles, which indicates the NLNFMB cathode possibly undergoes a distinct electrochemical reaction. The differential capacity analysis (dQ dV^−1^) plots of two cathodes were examined to further understand the electrochemical performances (Supplementary Fig. 6a, b). Two predominant redox peaks are observed for the NLNFM and NLNFMB cathodes: the peaks in low voltage region below 3 V are ascribed to O3−P3 phase transition, and the other peaks in high voltage region above 4.2 V are attributed to the P3−OP2 phase transition as discussed later. In addition, for NLNFM, an obvious oxidation peak at about 4.1 V in the first cycle shows up, without the corresponding reduction peak (Supplementary Fig. 6a). The absence indicates that the plateau about 4.1 V in charge/discharge curve is irreversible. In view that the electrolyte with 1 M NaPF_6_ in carbonate solvents is electrochemically stable within the operation voltage of the cathode^41^, the extra capacity contribution of the NLNFM cathode could come from the irreversible oxygen redox reaction, which is similar to the lithium-rich layered oxides^42,43^, and further confirmed by operando differential electrochemical mass spectrometry. Compared with the previously reported compound Na_0.85_Li_0.1_Ni_0.18_Fe_0.18_Mn_0.54_O_2_ which solely undergoes the cationic redox as being charged to 4.5 V, our control material NaLi_1/9_Ni_2/9_Fe_2/9_Mn_4/9_O_2_ shows a complex Na storage electrochemistry that invites both cationic and anionic redox at a much lower voltage of 4.3 V. This result indicates that even a subtle variation in chemical composition may have a prominent influence on the structural evolution and electrochemical Na storage mechanism of layered oxide cathodes. After doping with light-weight boron for NLNFMB, no oxidation peak at 4.1 V is observed in the first cycle, which is well consistent with the smooth sloping GCD curves for NLNFMB. The B-substituted NLNFMB thus delivers much better capacity retention (76.3%) than that of B-free NLNFM (45.1%) after 80 cycles at 25 mA g^−1^ (Fig. 4c). Furthermore, at a specific current of 500 mA g^−1^ (Fig. 4d), the B-substituted NLNFMB cathode delivers an improved rate capability of 84.9 mA h g^−1^ compared with that of the unsubstituted NLNFM (35.6 mA h g^−1^), which is consistent with the results of lower energy barriers of Na^+^ diffusion as calculated above. In particular, the comparison of long cycling performance at 1 C (250 mA g^−1^) shows that the NLNFMB cathode maintains a much higher capacity retention of 82.8% after 200 cycles than that of the NLNFM cathode (66.2%) (Fig. 4e). The NLNFMB electrodes exhibit stable Coulombic efficiency compared to NLNFM electrodes (Supplementary Fig. 7). The aforementioned results confirm that the doping of boron is beneficial to suppress irreversible oxygen redox reaction, guarantee good structural integrity, and endow the NLNFMB with the good electrochemical performance upon Na^+^ intercalation/deintercalation process. What’s more, different content of boron doped into NLNFM were synthesized to check the optimal species. In Supplementary Fig. 8, XRD patterns show no impurities in NaLi_1/9_Ni_2/9_Fe_2/9_Mn_4/9_B_1/100_O_2_ (NLNFMB0.01) while some impurities with the small peaks around 17° appear in NaLi_1/9_Ni_2/9_Fe_2/9_Mn_4/9_B_1/25_O_2_ (NLNFMB0.04). The related electrochemical performance was showed in Supplementary Fig. 9. The NLNFMB0.02 exhibits the highest discharge capacity and capacity retention of 82.8% compared with 68.3% for NLNFMB0.01 and 82.3% for NLNFMB0.04. Meanwhile, the corresponding Coulombic efficiency in Supplementary Fig. 10 shows the good reversibility of NLNFMB0.02. The electrochemical performance of hard carbon (HC)||NLNFMB full cells was further evaluated. The initial first GCD curve at 1C (250 mA g^−1^) shows that the full cell delivers a discharge capacity of 133.9 mA h g^−1^ based on the mass of cathode active material with the average voltage of 3.1 V (Fig. 5a), thus generating a specific energy of up to 224 Wh kg^−1^ based on the mass of NLNFMB and HC active material^44,45^. At a specific current of 1C (250 mA g^−1^) (Fig. 5b), the full cell retains a high capacity of 80.1% after 100 cycles and a stable Coulombic efficiency (Supplementary Fig. 11). A comparison with other Na-ion full cells reported in the literature is disclosed in Supplementary Table 9^46–48^. Supplementary Fig. 12 shows a 71.28 mAh single-coated lab-scale Na-ion pouch cell comprising a pre-sodiated hard carbon-based anode and the pouch cell with B-doped cathode material could power light-emitting diode lamps as proof of concept.

### Structural evolution

**Fig. 6:**
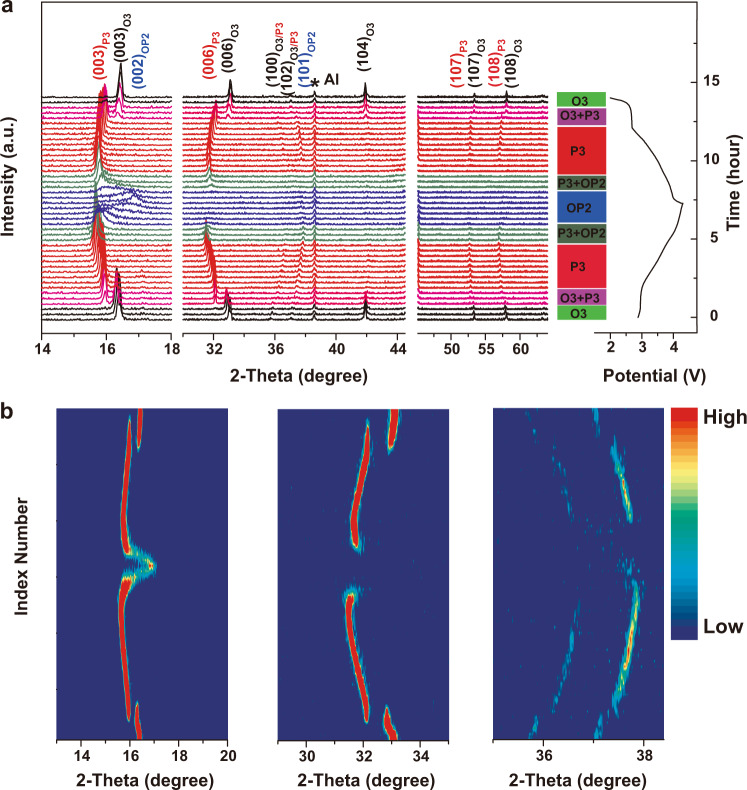
Structural evolution of NLNFMB cathode material in half-cell configuration. In situ XRD patterns during the first charge/discharge of NLNFMB at a current rate of 0.1C (25 mA g^−1^) between 2.0 and 4.3 V (**a**). Contour plot of main peak evolution (**b**).

To further investigate the structural evolution of NLNFMB, in situ XRD patterns and corresponding intensity contour maps were recorded upon Na^+^ intercalation/deintercalation process of the first cycle. During the charging process (Fig. 6), the (003) and (006) peaks that are associated with the pristine O3 phase shift to a lower angle. The shift indicates the expansion of *c* axis due to the increased electrostatic repulsion between adjacent oxygen layers. With charging to about 3.0 V, an additional (003) XRD diffraction peak gradually appears at a lower angle. The appearance reflects the formation of the P3 phase, indeed consistent with the platform of the GCD curve in the voltage range between 2.9 and 3.0 V. When the cell was continuously charged to 4.0 V, the diffraction peaks associated with the O3 phase completely disappear, and the P3 phase is preserved. Upon the further extraction of Na ions, a new phase OP2 appears, accompanied by the reversible phase transformation of P3 phase, similar to the structural evolution of O3-type NaFe_0.2_Mn_0.4_Ni_0.4_O_2_^49^. Upon further charging to 4.3 V, the (002) peak of OP2 phase shifts to higher angles, the contraction is well related to the oxidation of O^2−^ and the resultant smaller repulsion, as reported early for LiCoO_2_ or Ni-rich Li-based layered oxides^50,51^. During the subsequent discharge process, the NLNFMB electrode undergoes an exact opposite evolution of OP2–P3–O3, suggesting the highly reversible phase transformation during Na^+^ intercalation/deintercalation process. By contrast, the B-free NLNFM electrode exhibits similar but different structural changes in Supplementary Fig. 13: during the charging process, the NLNFM electrode undergoes a same phase evolution of O3–P3–OP2, the *c* parameter increases first and then decreases for both electrodes while *a* parameter decreases first and then increases during the charging process. Note that the lattice parameters change Δ*c* for NLNFMB is 4.8% slightly smaller than that of NLNFM (5.7%), indicating the reduced lattice volume change and more stable structure of NLNFMB in Supplementary Fig. 14. A small amount of the P3 phase still remains at the end of discharging (Supplementary Fig. 13a, b), namely, the O3 and P3 phase co-exist after one charge/discharge process. After the completion of 200 cycles, the NLNFM structure is converted completely from the initial O3 phase to the P3 phase, whereas the NLNFMB sample still maintains the perfect O3 phase (Supplementary Fig. 15a, b). We attribute the appearance of the P3 phase for NLNFM to the fact that the oxygen vacancies produced by oxygen loss result in the difficulty in re-insertion of Na^+^ into lattices. Therefore, the B-doping reduces the variation of lattice parameters and facilitates the reversible O3−P3−OP2 phase transformation of NLNFMB, stabilizing the oxygen redox reaction and hence ensuring structural stability.

### Charge compensation mechanism

**Fig. 7:**
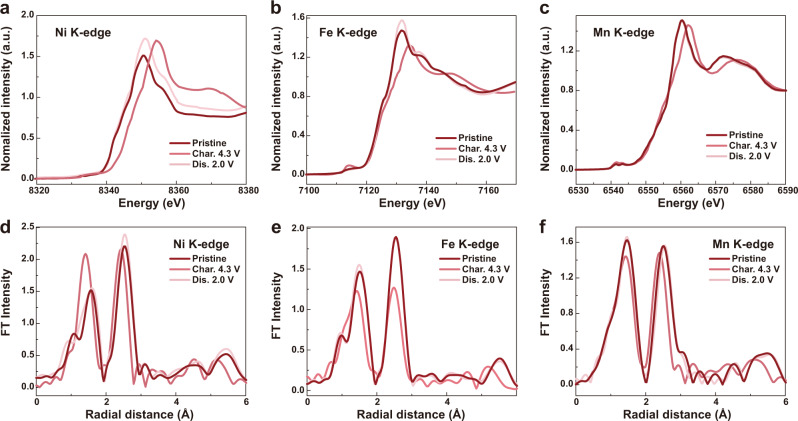
Charge compensation mechanism upon at the first cycle in half-cell configuration. Ex situ XANES spectra at the Ni (**a**), Fe (**b**), Mn (**c**) K-edge of NLNFMB at different charge/discharge states. Corresponding ex situ EXAFS spectra at the Ni (**d**), Fe (**e**), Mn (**f**) K-edge of NLNFMB electrodes at different charge/discharge states.

Ex situ X-ray absorption spectroscopy (XAS) and ex situ XPS were used to probe the reaction mechanism during the charge/discharge process. X-ray absorption near edge structure (XANES) comparison between NLNFM and NLNFMB shows a slight shift of Fe and Mn absorption edge to lower energy after B substitution (Supplementary Fig. 16a–c). The shift indicates that Fe and Mn are reduced to lower valence, corroborating the successful B-doping into the structure. The lower valences of Fe and Mn are ascribed to the fact that B atom is surrounded by Li–Fe–Mn, which has been discussed in the computational part. In addition, the lower valence of transition metal (TM) is responsible for the higher capacity of NLNFMB. Figure 7a–c exhibits the normalized Ni, Fe, and Mn K-edge XANES spectra of NLNFMB sample at different charge/discharge states. The Ni K-edge XANES spectra show a significant shift towards higher energy (∼3.4 eV) when charged to 4.3 V, which is larger than Ni^2+^ to Ni^3+^ redox change (∼2 eV) but smaller than Ni^2+^ to Ni^4+^ redox change (∼4 eV), indicating the redox reaction from Ni^2+^ to Ni^3+^ and partly oxidized to Ni^4+^ ^15,52^. When discharged to 2.0 V, the Ni K-edge XANES spectra recover to the initial position, suggesting that the Ni redox is electrochemically reversible. Similarly, the Fe K-edge XANES spectra shows slight but obvious shift towards higher energy when charged to 4.3 V, which demonstrates that Fe is oxidized to a higher valence and also return to the pristine position upon further discharge. Moreover, the Mn K-edge XANES spectra show a slight shift when the NLNFMB was charged to 4.3 V, and recover to the initial as discharged to 2.0 V. However, only few Mn involves the charge compensation, albeit the local environment of Mn center could sensitively influence the shape of Mn K-edge spectra^53^. This is because that the B substitution has a weaker influence on Mn than on Fe (Supplementary Fig. 16). In the case of NLNFM, the Ni and Mn K-edge XANES spectra show the evolution similar to that of NLNFMB (Supplementary Fig. 17); while the Fe K-edge XANES spectra show an inappreciable shift towards the higher energy as charged to 4.3 V. The result reflects that few Fe involves the charge compensation during the charging process. Furthermore, the energy shift of Fe K-edge in NLNFMB is about ~0.9 eV, larger than that of NLNFM (0.5 eV), suggesting more Fe participate in charge compensation for NLNFMB than for NLNFM (Supplementary Fig. 18). And ex situ XPS was carried on to investigate the Fe 2*p* peaks in layered oxide electrode charged to 4.3 V (Supplementary Fig. 19). The peak area at 715.3 eV of NLNFMB is larger than that of NLNFM, indicating more Fe^3+^ were oxidized to Fe^4+^. Therefore, more Fe participates in charge compensation for NLNFMB than for NLNFM, thus favoring the high reversible capacity of NLNFMB.

Further comparison of extended X-ray absorption fine structure (EXAFS) spectra at Ni, Fe, and Mn K-edge are made between NLNFMB and NLNFM to gain the local coordination information^54^. The significant changes of EXAFS peak intensity in discharge cutoff state and initial state for NLNFM indicate the obvious structural changes (Supplementary Fig. 17). Figure 7d–f shows that the intensity of both TM–TM and TM–O coordination shells of NLNFMB sample returns to the initial state upon discharging to 2.0 V. However, this is not the case for the B-free NLNFM sample after the completion of discharging to 2.0 V. All the aforementioned results suggest that the NLNFMB possesses the good structural stability during the charge/discharge process, which is consistent with the reversible phase transition of NLNFMB observed by in situ XRD.

XPS technique was used to probe the oxygen redox reaction species (Supplementary Figure 20). A pronounced peak at 530.9 eV is visible for the NLNFMB and NLNFM when charged to 4.3 V, which is ascribed to the oxidation of O^2−^ with the formation of peroxo-related species (O_2_^*n*−^)^4^. The presence informs that the two samples both undergo oxygen redox reaction. Comparison of O1*s* spectrum between the NLNFM and NLNFMB samples shows the larger area and higher intensity of O1*s* spectrum around the higher binding energy region of NLNFM. This indicates the high-amount decomposition species of electrolyte, thus forming the thick and resistive CEI and incurring the poor rate capability. The peak at 530.9 eV is invisible when discharged to 2 V, demonstrates the reduction of released oxygen species, and peroxo-related species (O_2_^*n*−^) at 530.9 eV appears again at the 2nd cycle, demonstrating the reversible oxygen redox in NLNFMB (Supplementary Fig. 21).

**Fig. 8:**
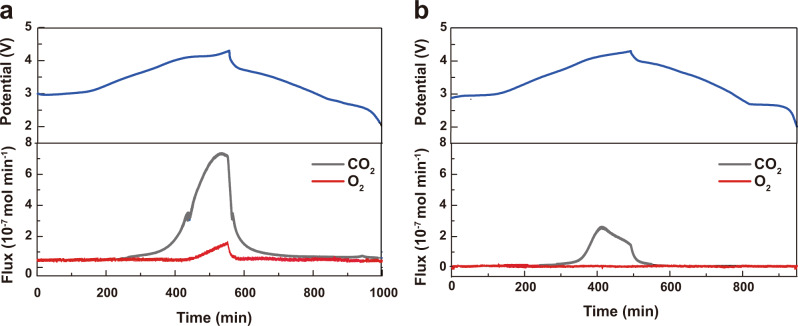
Gas loss of NLNFM and NLNFMB during (de)sodiation. DEMS data collected during the first cycle of NLNFM (**a**) and NLNFMB (**b**).

To further check the oxygen release from lattice due to the irreversible oxygen redox reaction, differential electrochemical mass spectrometry (DEMS) was used during Na^+^ intercalation/deintercalation process. The evolution of both O_2_ and CO_2_ is clearly detected during the charging of NLNFM (Fig. 8a, b). The detected CO_2_ gas is associated with the decomposition of a small amount of Na_2_CO_3_ and electrolyte oxidation when charged to the high voltage range from 3.5 V to 4.3 V^10^, and the formed O_2_ gas during charging to high voltage region is well related to the irreversibility of oxygen redox reaction resulting from oxygen loss from the lattice, responsible for the anomalously large capacity at the first charge of the initial GCD curve. In addition, the continuous O_2_ release was detected in the subsequent cycles upon charging the NLNFM sample (Supplementary Fig. 22). This pronounced oxygen release from lattice upon repeated cycles is also responsible for the above-mentioned capacity fading and the structural instability of the NLNFM during the cycling. Eventually, the NLNFM undergoes the irreversible structural transformation from the co-existing O3/P3 phases after one charge/discharge procedure (as evidenced by in situ XRD patterns), to the only P3 phase after the completion of 200 cycles (as shown by ex situ XRD patterns in Supplementary Fig. 15a). In marked contrast to NLNFM, the NLNFMB exhibits a completely different picture for the DEMS curve that no oxygen release evolved but instead only a small quantity of CO_2_ detected (Fig. 8b). The result strongly suggests that the incorporation of boron ions indeed suppresses the irreversible oxygen redox reaction, thus endowing the smooth GCD curve above 4.1 V and improving the structural stability. We estimated the capacity contribution provided by cationic redox and anionic redox in the first cycle based on Ni and Fe K-edge XANES spectra (Supplementary Fig. 23). We can see that the NLNFM delivers a low capacity from cationic redox (120 mAh g^−1^) and a larger capacity from anionic redox (77 mAh g^−1^), whereas the B-doped NLNFM shows a higher capacity from cationic redox (130 mAh g^−1^) and slightly lower capacity from anionic redox (43 mAh g^−1^). This indicates our B-doping strategy effectively suppresses the irreversible oxygen loss and facilitates more cationic redox concurrently.

## Discussion

In summary, we have demonstrated an improvement strategy of doping light-weight boron ions into the NLNFM lattices to effectively stabilize the reversible oxygen redox at charging high voltage region. Each B–O bond can accommodate extra negative valence change to O atom due to the strong covalent bond of boron with oxygen, which is conducive to alleviate the oxidation of O for charge compensation and successfully suppresses the irreversible oxygen redox as proved by DEMS. In particular, the boron doping promotes the reversible phase transition evidenced by in situ XRD. Therefore, B substitution facilitates the high reversibility of oxygen redox reaction and retains good structure stability, thus boosting the excellent cycling stability. In the meantime, B-doping could enhance Na-ion diffusion kinetics, resulting in a good rate performance. Another bonus brought by B-doping is a more cationic redox reaction. As a result, the NLNFMB electrodes show a reversible capacity of 160.5 mA h g^−1^ at 25 mA g^−1^ and a reversible discharge of 120 mA h g^−1^ at 250 mA g^−1^ with excellent cycling stability (82.8% after 200 cycles). This light-weight B substitution provides a feasible method to suppress the detrimentally irreversible oxygen release with a positive capacity contribution. The alternative light-weight elements (P, Si) with strong oxygen binding are expected to stabilize the oxygen redox and realize the high-capacity cathodes for NIBs and LIBs as well.

## Methods

### Materials preparation

The NaLi_1/9_Ni_2/9_Fe_2/9_Mn_4/9_O_2_ (NLNFM) and NaLi_1/9_Ni_2/9_Fe_2/9_Mn_4/9_B_1/50_O_2_ (NLNFMB) compounds were synthesized by simple solid-state reactions. The stoichiometric mixtures of Na_2_CO_3_ (99.5%; Alfa Aesar), Li_2_CO_3_ (99.5%; Alfa Aesar), Fe_2_O_3_ (99.9%; Alfa Aesar), Mn_2_O_3_ (98%; Alfa Aesar), and B_2_O_3_ (99.9%, Alfa Aesar) were ground and then calcined at 950 °C for 15 h, after cooling to room temperature, and transferred to an argon-filled glove box immediately.

### Materials characterization

X-ray powder diffraction was collected in a Bruker D8 Advance diffractometer with Cu Ka radiation (*λ* = 1.5418 Å). The TOPAS software s was utilized to analyze the crystal structure based on the Rietveld refinement method. A specially designed Swagelok cell was equipped with an aluminum window for X-ray penetration for in situ XRD experiment. The specific ratios of the samples were measured by inductively coupled plasma atomic emission spectrometry (ICP-AES; Thermo Icap 6300). The sample was dissolved by aqua regia and measured with the diluted solvent. The morphologies and microstructures were characterized by using scanning electron microscopy (SEM; JEOL JSM-6701F). The HRTEM image and EDS maps were examined by TEM (JEM; 2100F). XPS measurements were carried out with an AXIS Supra using an Al Ka achromatic X-ray source. XAS measurements were carried out at beamline BL14W1 of the Shanghai Synchrotron Radiation Facility (SSRF), China, operating with a Si (111) double-crystal monochromator. The OmniStar GSD 320 instrument with QMA 200M analyzer and C-SEM/Faraday detector was used to collect the differential electrochemical mass spectrometry (DEMS) spectra. The SIMS analysis was performed with a ToF-SIMS 5 instrument (ION-ToF GmbH, Münster, Germany) equipped with a 30 keV Bi^3+^ primary ion gun and a 2 keV Cs^+^ sputter gun. An electron flood gun was used for charge neutralization.

### Electrochemical characterizations

The electrochemical performance of NLNFM and NLNFMB compounds were completed in CR2032 coin-type cells and collected on an Arbin BT2000 system. The working electrode consists of 80 wt.% active materials, 10 wt.% conductive carbon (super P), and 10 wt.% polyvinylidene fluoride (PVdF) binder. The prepared slurry was cast onto an Al foil and dried at 80 °C overnight under a vacuum. The areal mass loading of active material on the cathode was ~3.5 mg cm^−2^. Na half cells based on CR2032 coin-cell configuration were assembled in an argon-filled glove box (H_2_O ≤ 0.1ppm; O_2_ ≤ 0.1ppm) by pairing the cathode with a Na-metal disc (99.8%; Alfa Aesar) as the anode. Both electrodes had a diameter of 10 mm, and were separated by a Whatman glass fiber (diameter: 20 mm), on which 180 μL of electrolyte was added to ensure complete wetting of both electrodes. The electrolyte consisted of 1 M NaPF_6_ in ethylene carbonate (EC) and diethyl carbonate (DEC) (1:1 in volume; with the addition of 5 vol.% fluoroethylene carbonate, FEC; H_2_O ≤ 20 ppm). The charge/discharge data were collected between 2.0 and 4.3 V at various rates under 25 °C. For the full cells, the active materials of the anode are hard carbon (HC) and the HC electrode consists of 80 wt.% active materials, 10 wt.% conductive carbon (super P), and 10 wt.% PVdF binder. We assembled two kinds of full cells, e.g., 2032 coin cell, and pouch cell with a single-layer configuration. The electrodes are single side coated. The HC anode was pre-sodiated with Na metal and subsequently paired with the NLNFMB cathode at a N/P ratio of 1.2. Galvanostatic charge/discharge measurements of coin cells were performed under 25 °C between 1.5 and 4.2 V at 1C (1C = 250 mAh g^−1^ based on the cathode specific capacity in the half-cell test). In the pouch cell, the area of the cathode is 71.28 cm^2^ with designed areal capacity of 1 mAh cm^−2^ and the areal mass loading of the HC anode is 4.2 mg cm^−2^. The overall capacity of the pouch cell in Fig. 5b is 71.28 mAh. The pouch cell was used to power the light-emitting diodes with the logo of “ICCAS” at full charge state.

### Specific energy calculation

According to the formula, *E*_C_ = *C*_C_ * *V*_C_, where *E*_C_ denotes the specific energy, *C*_C_ denotes the gravimetric-specific capacity of the cathode material and V_C_ denotes the average voltage of the half cell (which approximates the average cathode potential vs. Na^+^/Na). In this way, a specific energy of 521 Wh kg^−1^ based on the mass of cathode material can be calculated for Na||NLNFMB coin cell (*C*_C_ = 160.5 mAh g^−1^; V_C_ = 3.25 V). The specific energy of the full cell was calculated based on the cathode specific capacity (133.9 mAh g^−1^), the average voltage of the full cell (*V*_C_ = 3.1 V), and a N/P ratio of 1.2 adopted for the cell manufacture, which gave a value of 224 Wh kg^−1^.

### Computational details

All first-principle calculations were performed in spin-polarized mode with density functional theory (DFT) by the projector-augmented wave (PAW) method implemented in the VASP package^55^. The generalized gradient approximation as developed by Perdew, Burke, and Ernzerhof (PBE)^56^ is employed. Hubbard U potential correction was applied to take into account the self-interactions of on-site d-orbital in Mn (3.9 eV), Fe (4.0 eV), Ni (6.0 eV) atoms^57^. The kinetic energy cutoff 400 eV and a Γ-centered 2 × 2 × 1 k-mesh in the Brillouin zone have been utilized for the convergence of total energy with a criterion of 1 × 10^−4^ eV per atom.

The O vacancy formation energy for the B-doped and undoped materials was calculated based on the following formula: *E*_f,Ovac_ = *E*_ovac_ − *E*_pristine_ + 1/2*E*o_2_, where *E*_f,Ovac_ is the formation energy of O vacancy, *E*_Ovac_ and *E*_pristine_ are the energy of lattice structures with and without the O vacancy, and *E*o_2_ is the calculated energy for O_2_ molecule. The capacity change ratio was calculated based on the formula: $\frac{{{{{{\rm{Capacity}}}}}}\; {{{{{\mathrm{after}}}}}}\; {{{{{\mathrm{doping}}}}}}-{{{{{\rm{capacity}}}}}}\; {{{{{\mathrm{before}}}}}}\; {{{{{\mathrm{doping}}}}}}}{{{{{{\rm{Capacity}}}}}}\; {{{{{\mathrm{before}}}}}}\; {{{{{\mathrm{doping}}}}}}}$.

## Supplementary information

Supplementary information

## Data Availability

The data that supporting the findings of this study are available from the corresponding author on reasonable request.
