# Supplementary material for: Boron-doped sodium layered oxide for reversible oxygen redox reaction in Na-ion battery cathodes
Source: Nat Commun. 2021 Sep 6;12:5267. doi: 10.1038/s41467-021-25610-7 (PMC8421359; doi:10.1038/s41467-021-25610-7)
Supplement: Supplementary file 1 — Supplementary information [file 41467_2021_25610_MOESM1_ESM.pdf]

Supplementary information for

Boron-doped sodium layered oxide for reversible oxygen redox reaction in Na-ion battery cathodes

Yu-Jie Guo[1,2‡], Peng-Fei Wang[1,2‡], Yu-Bin Niu[1,2‡], Xu-Dong Zhang[1,2], Qinghao Li[2,3], Xiqian Yu[2,3], Min Fan[1,2], Wan-Ping Chen[1,2], Yang Yu[2], Xiangfeng Liu[2], Qinghai Meng[1,2], Sen Xin[1,2], Ya-Xia Yin[1,2]* and Yu-Guo Guo[1,2]*

[1]CAS Key Laboratory of Molecular Nanostructure and Nanotechnology, CAS Research/Education Center for Excellence in Molecular Sciences, Beijing National Laboratory for Molecular Sciences, Institute of Chemistry, Chinese Academy of Sciences (CAS), Beijing 100190, P. R. China. [2]University of Chinese Academy of Sciences, Beijing 100049, P. R. China. [3]Beijing National Laboratory for Condensed Matter Physics, Institute of Physics, CAS, Beijing 100190, P. R. China.

‡ These authors contributed equally to this work.

* To whom correspondence should be addressed.

E-mail: yxyin@iccas.ac.cn; ygguo@iccas.ac.cn

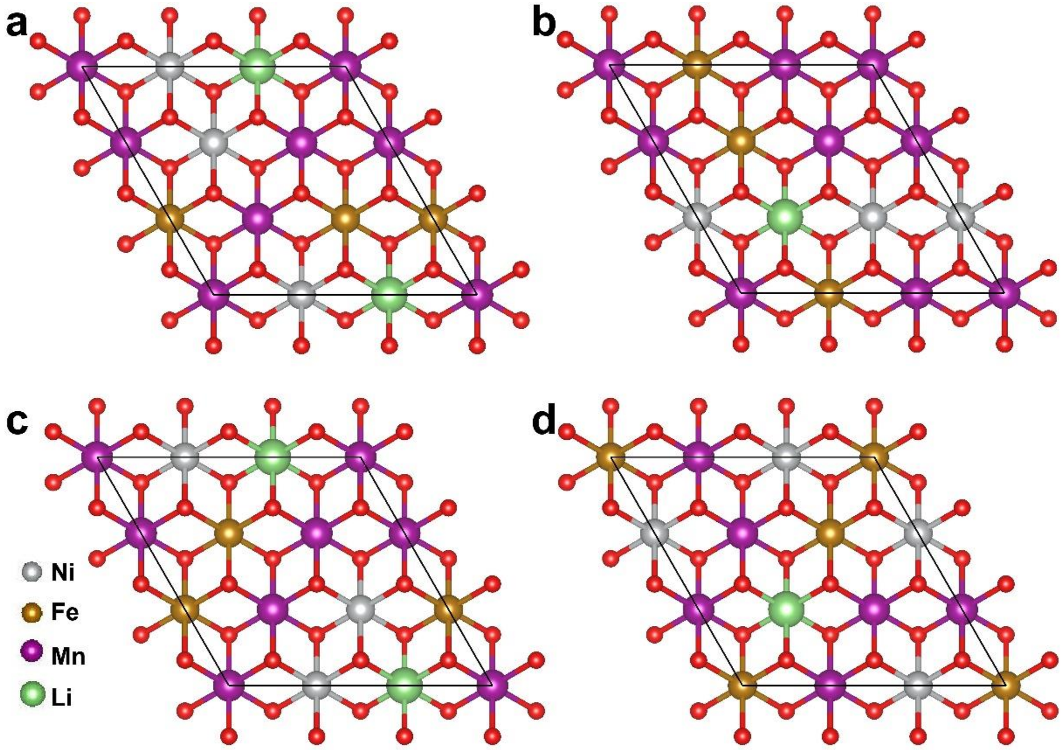

Supplementary Figure 1. Structure of NLNFM. Li surrounding more Ni and Fe (**a**), Fe and Fe (**b**), Ni and Ni (**c**), Mn and Mn (**d**).

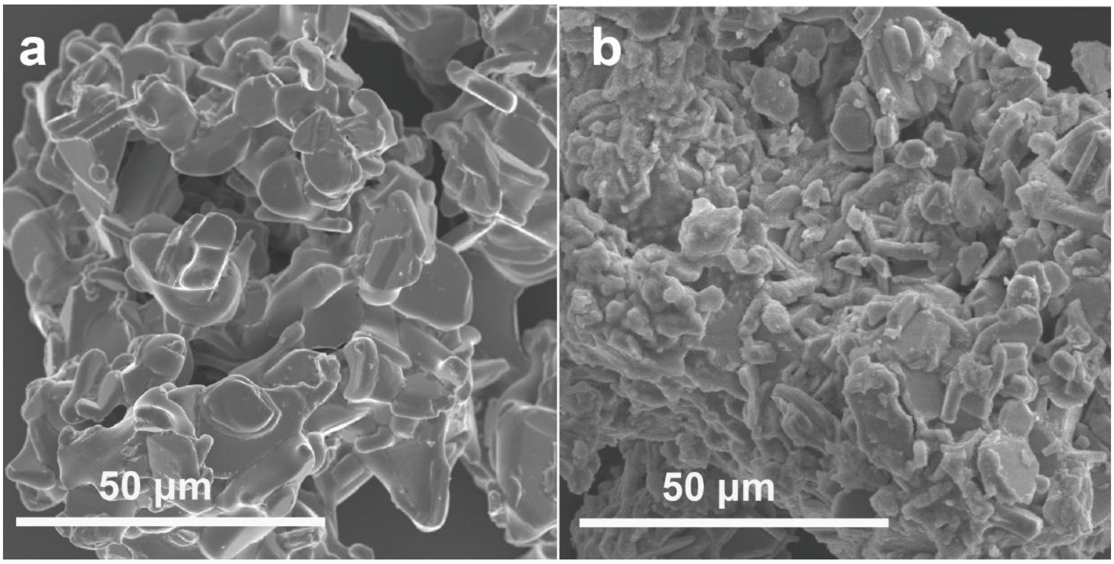

Supplementary Figure 2. SEM images of NLNFMB (**a**) and NLNFM (**b**) powders.

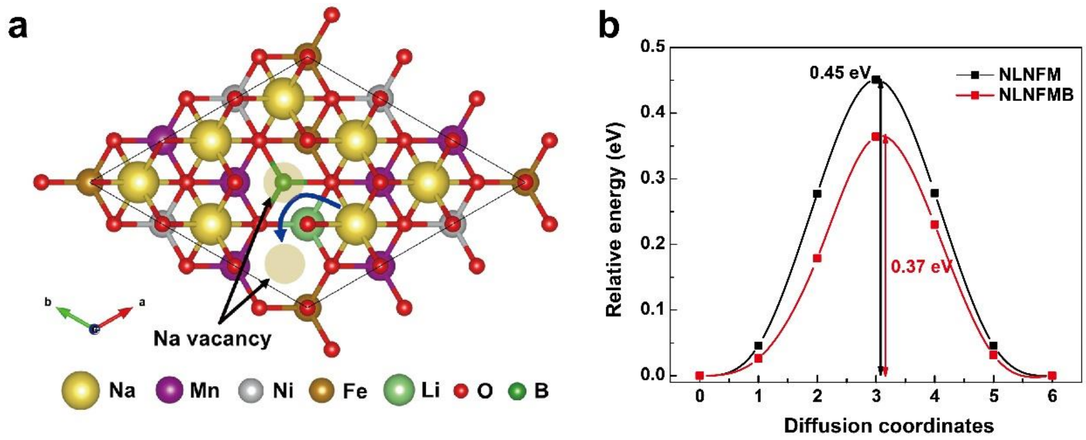

Supplementary Figure 3. Na-ion migration energy profile. Na-ion migration path (**a**) and migration energy barrier (**b**).

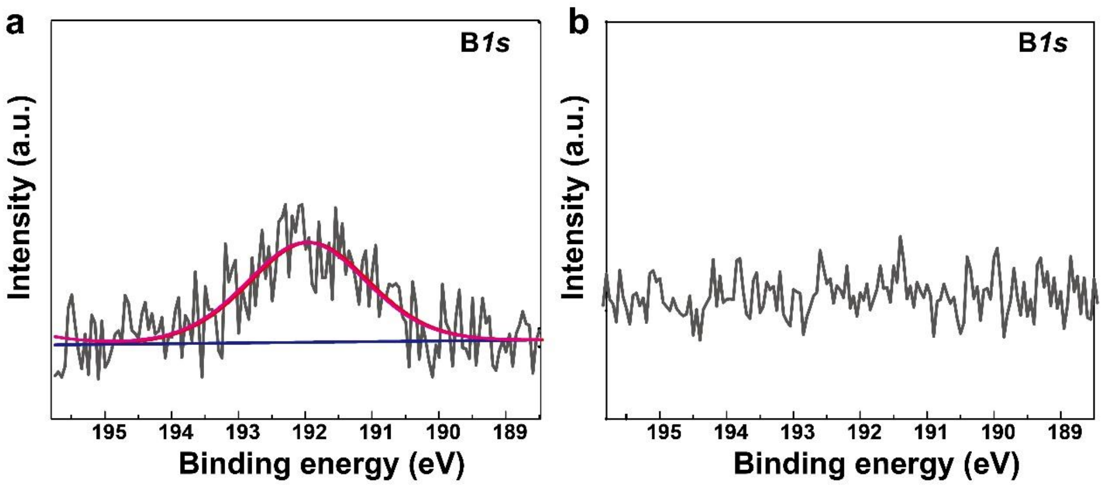

Supplementary Figure 4. XPS spectra of NLNFMB **(a)** and NLNFM **(b)** powders for B *1s* after Ar^+ etching 10 minutes.

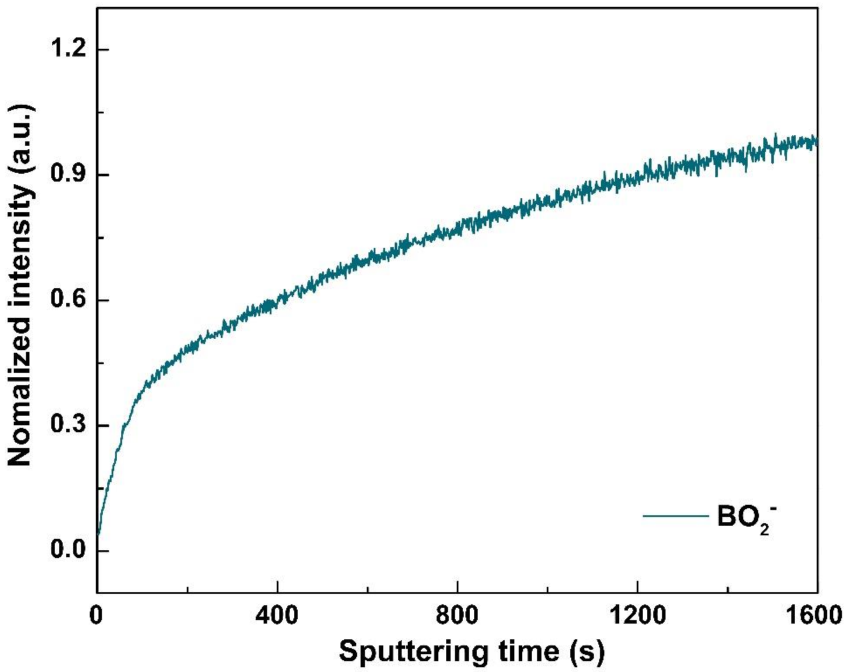

Supplementary Figure 5. ToF-SIMS depth analysis. ToF-SIMS depth profiles of secondary-ion fragments (negative mode, normalized to maximum) of NLNFMB electrode.

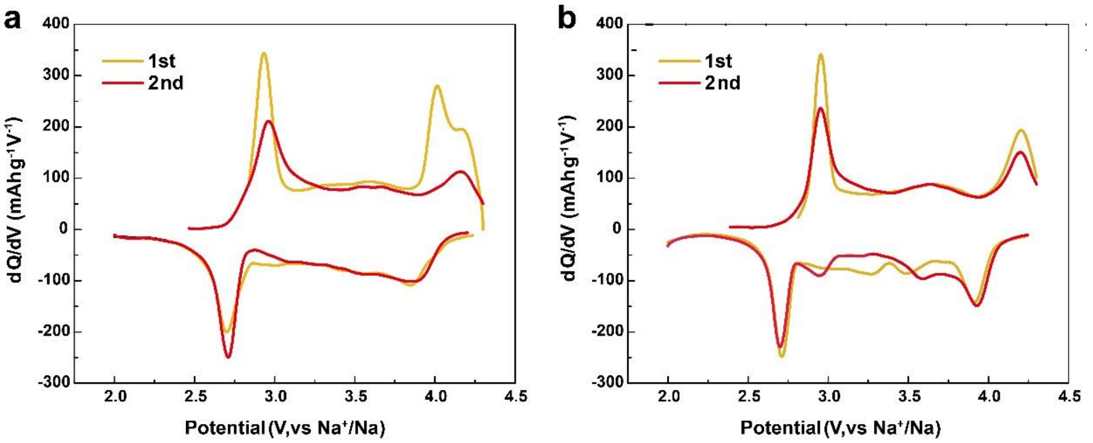

Supplementary Figure 6. dQ dV^{-1} profiles of NLNFM (**a**) and NLNFMB (**b**) electrodes.

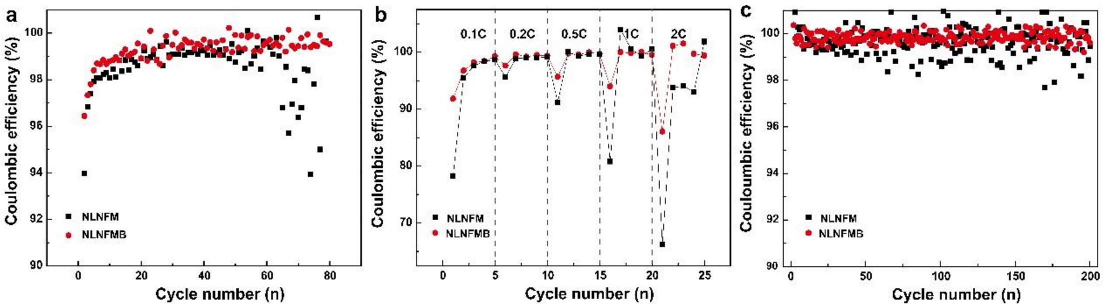

Supplementary Figure 7. Coulombic efficiency of NLNFM and NLNFMB electrodes cycled at 0.1C (25 mA g^{-1}) **(a)**, different rates **(b)**, and 1C rate (250 mA g^{-1}) **(c)** respectively.

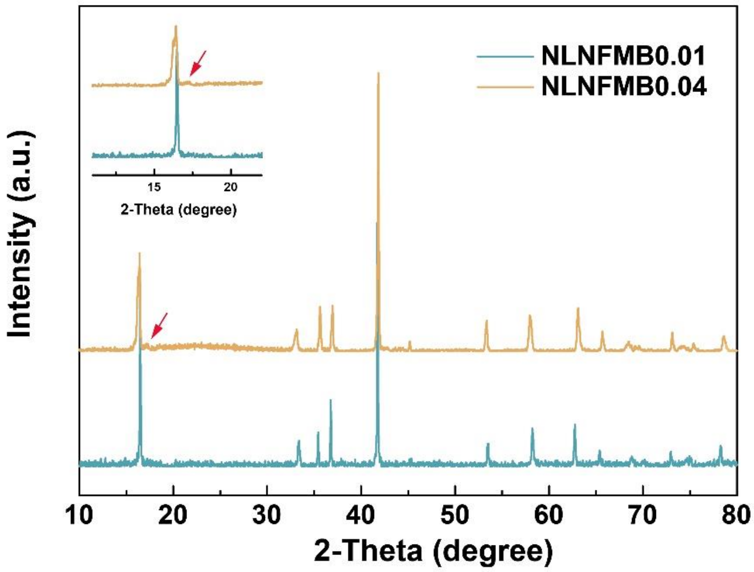

Supplementary Figure 8. Structural analysis. XRD patterns of NLNFMB0.01 and NLNFMB0.04 powders, respectively.

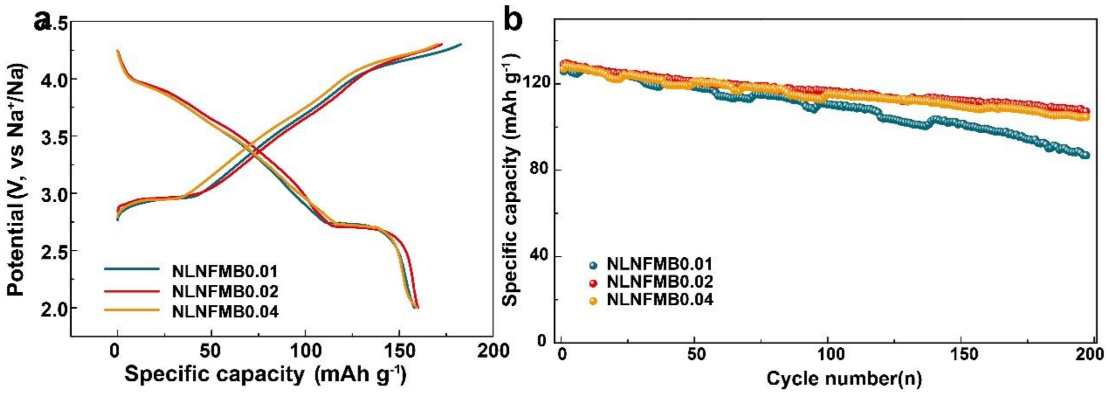

Supplementary Figure 9. Electrochemical properties of NLNFMB0.01, NLNFMB0.02 and NLNFMB0.04 electrodes in half-cell configuration. Galvanostatic charge/discharge voltage profiles at a 0.1C (1C = 250 mA g^{-1}) (**a**) and cycling performance at 1C rate between 2.0 and 4.3 V (**b**).

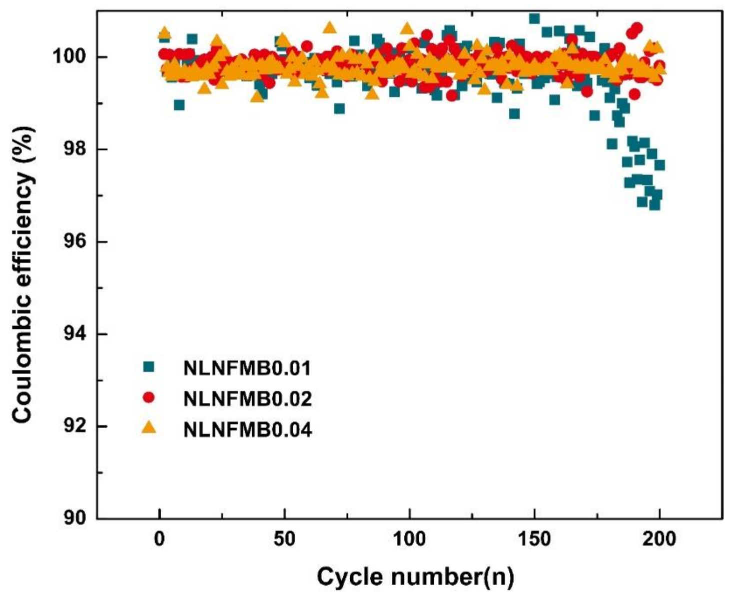

Supplementary Figure 10. Coulombic efficiency of NLNFM and NLNFMB electrodes cycled at 1C rate (250 mA g^{-1}).

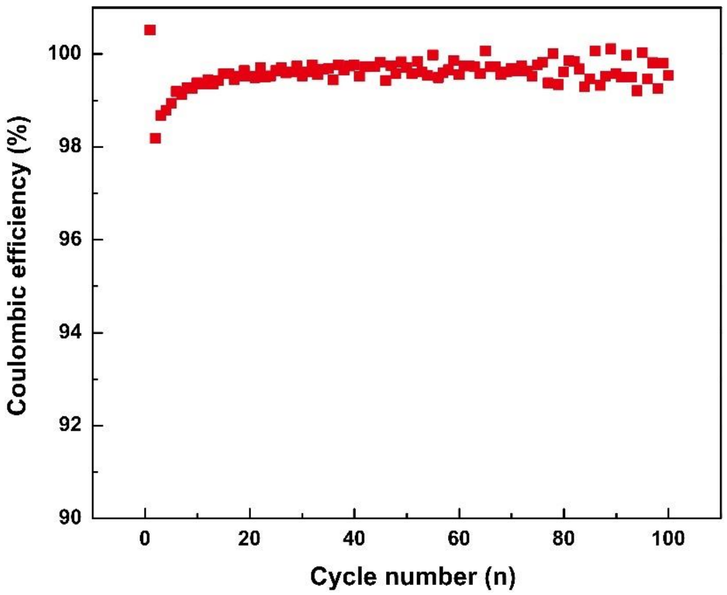

Supplementary Figure 11. Coulombic efficiency of NLNFMB electrode cycled at 1C rate (250 mA g^{-1}) in the full cell.

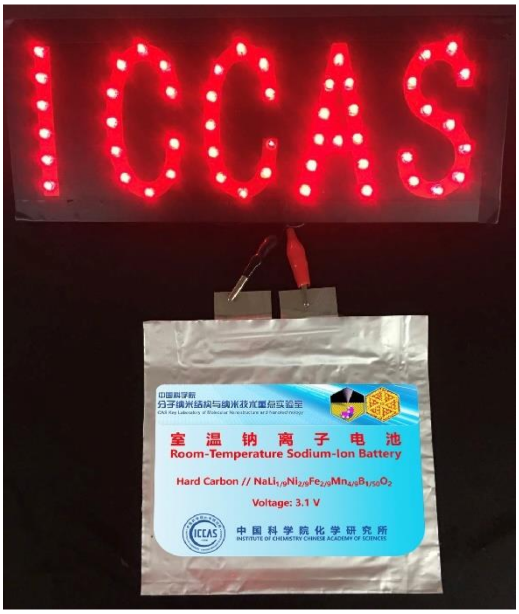

Supplementary Figure 12. Pouch cell of B-doped cathode to power light-emitting diode lamps as proof of concept.

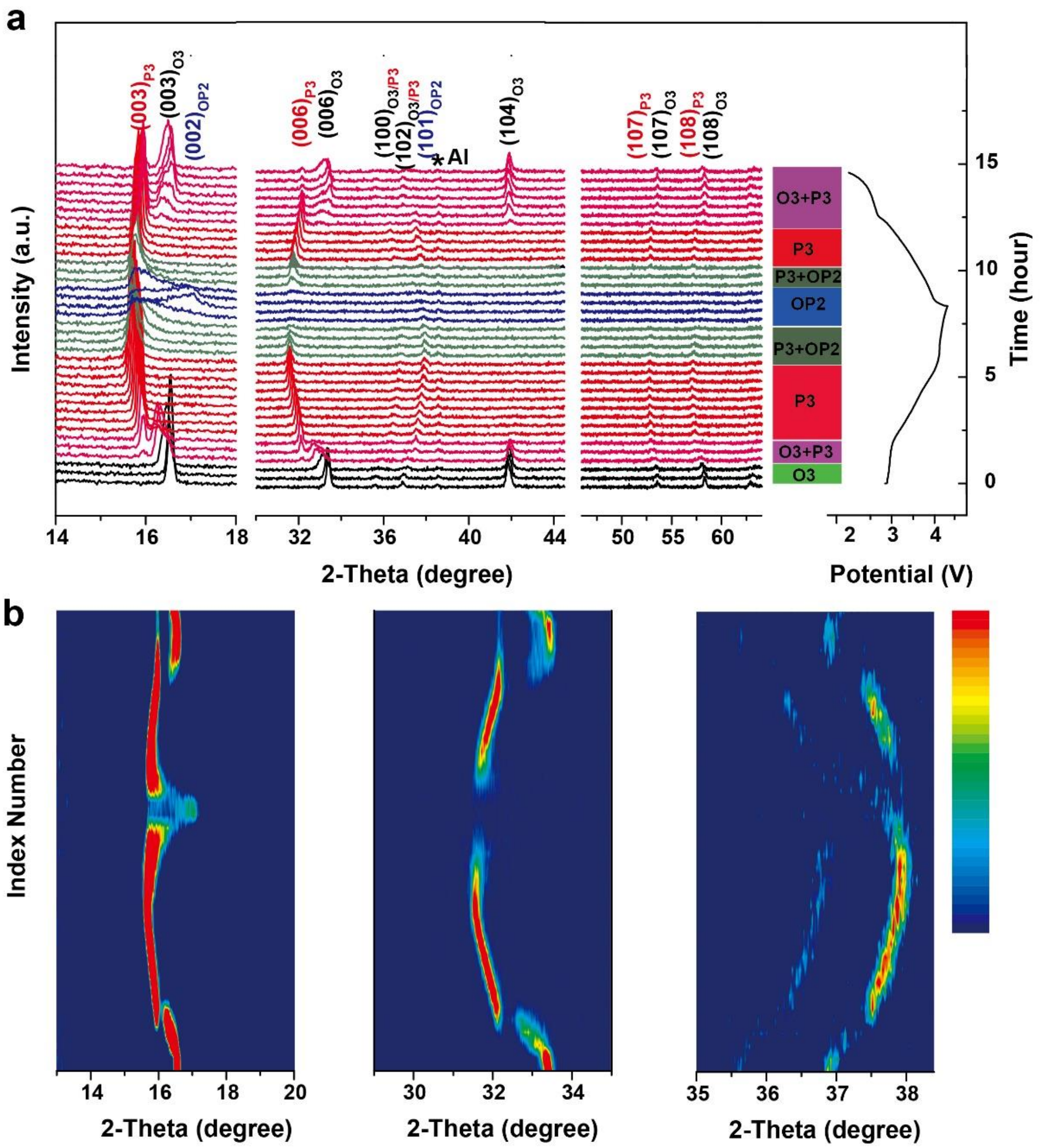

Supplementary Figure 13. Structural evolution in half-cell configuration. *In situ* XRD patterns during the first charge/discharge of NLNFM electrode at a current rate of 0.1C between 2.0 and 4.3 V (**a**) and contour plot of main peak evolution (**b**).

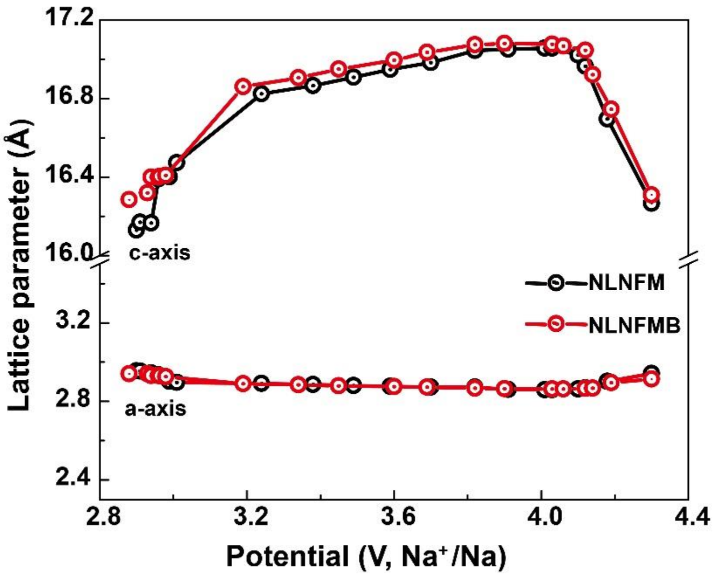

Supplementary Figure 14. Lattice parameter variation during first charging process.

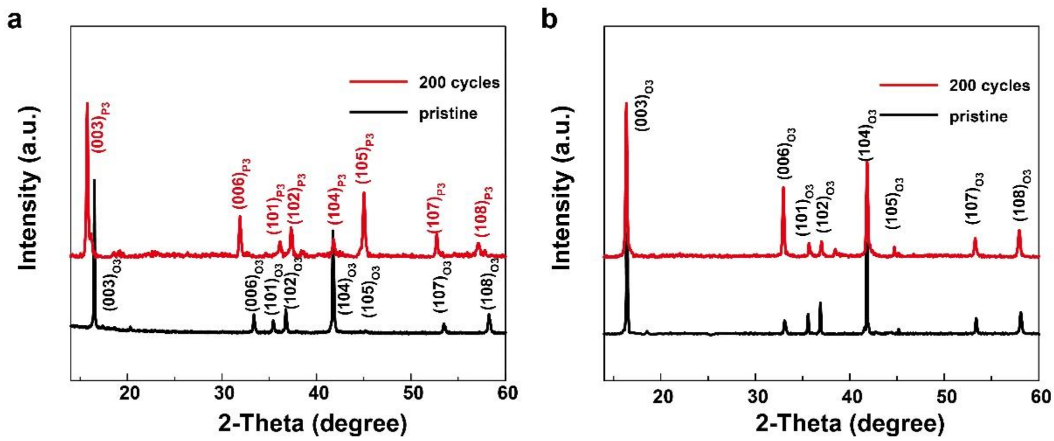

Supplementary Figure 15. *Ex situ* XRD patterns of the NLNFM (**a**) and the NLNFMB (**b**) electrodes before and after 200 cycles.

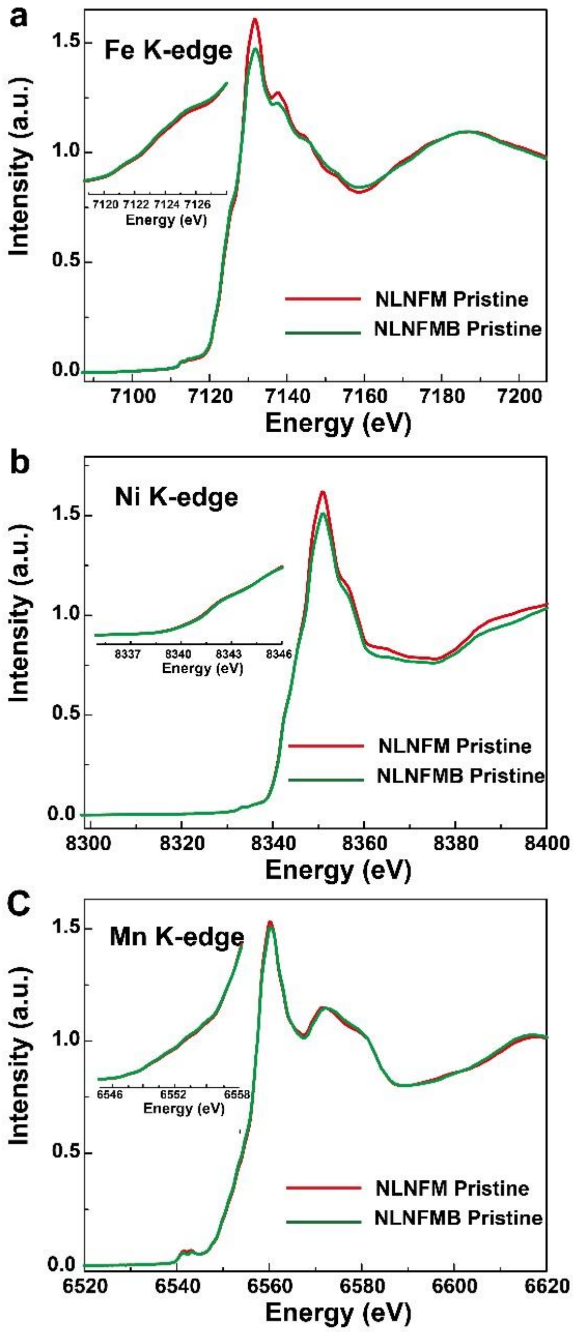

Supplementary Figure 16. XANES spectra at the Fe (**a**), Ni (**b**), Mn (**c**) K-edge of NLNFM and NLNFMB electrodes.

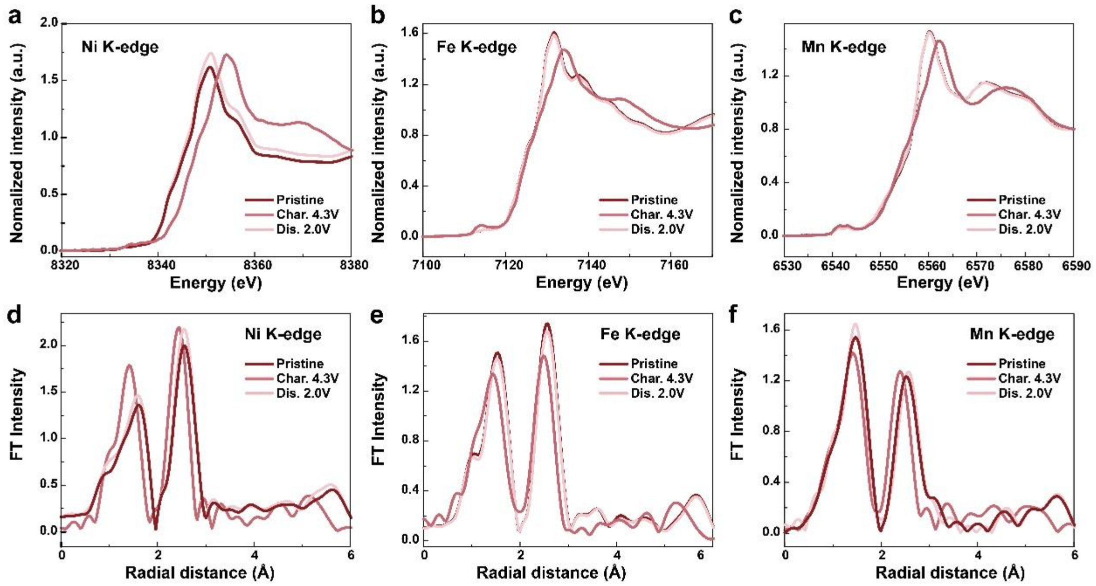

Supplementary Figure 17. *Ex situ* XANES spectra at the Ni (**a**), Fe (**b**), Mn (**c**) K-edge of NLNFM electrodes at different charge/discharge states. Corresponding *ex situ* EXAFS spectra at the Ni (**d**), Fe (**e**), Mn (**f**) K-edge of NLNFM electrodes at different charge/discharge states.

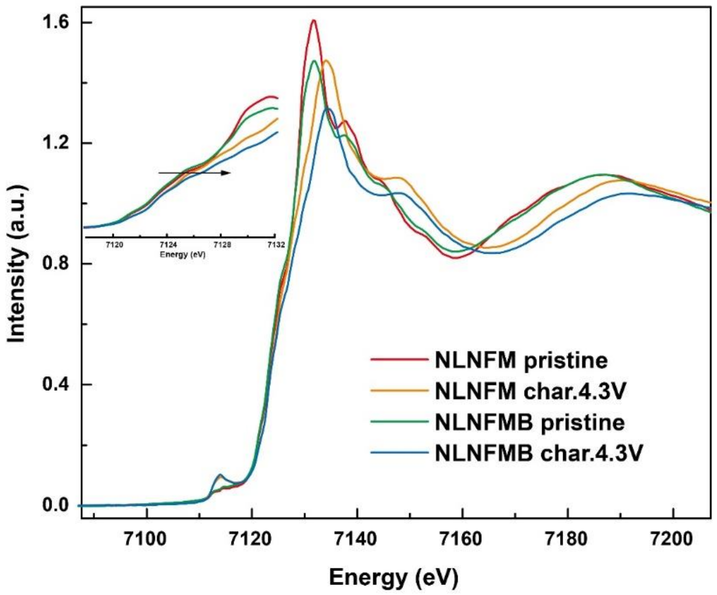

Supplementary Figure 18. Comparison of Fe K-edge XANES spectra of NLNFM and NLNFMB electrodes at different states.

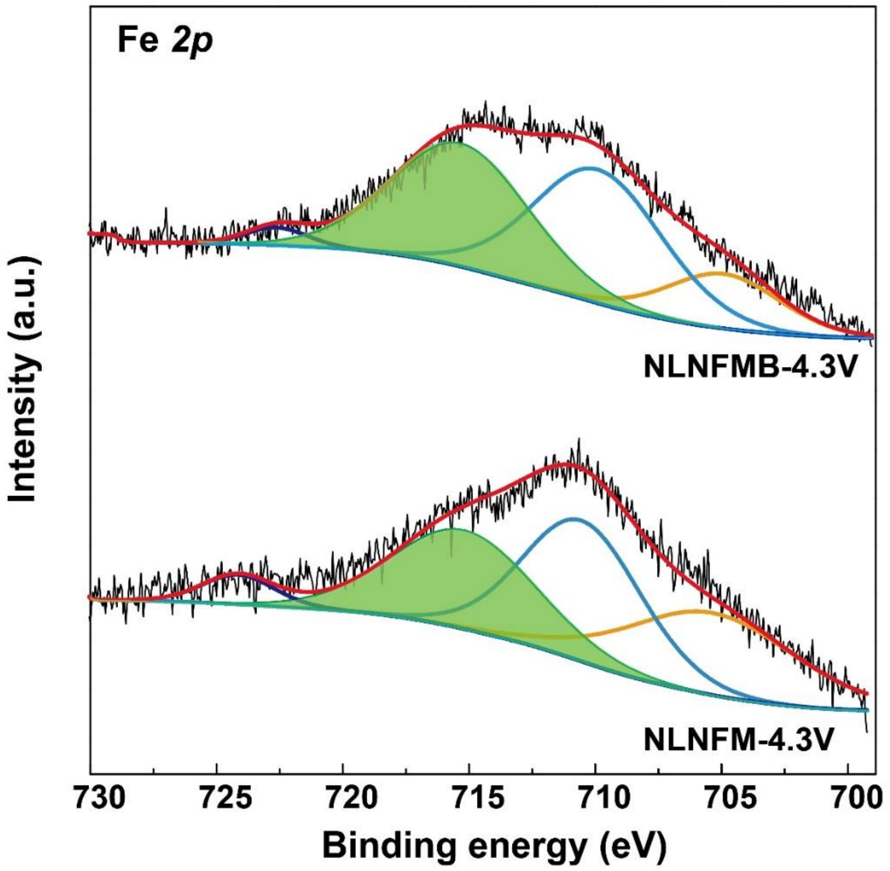

Supplementary Figure 19. *Ex situ* XPS spectra of Fe *2p* in NLNFM and NLNFMB electrodes at charging to 4.3V, respectively.

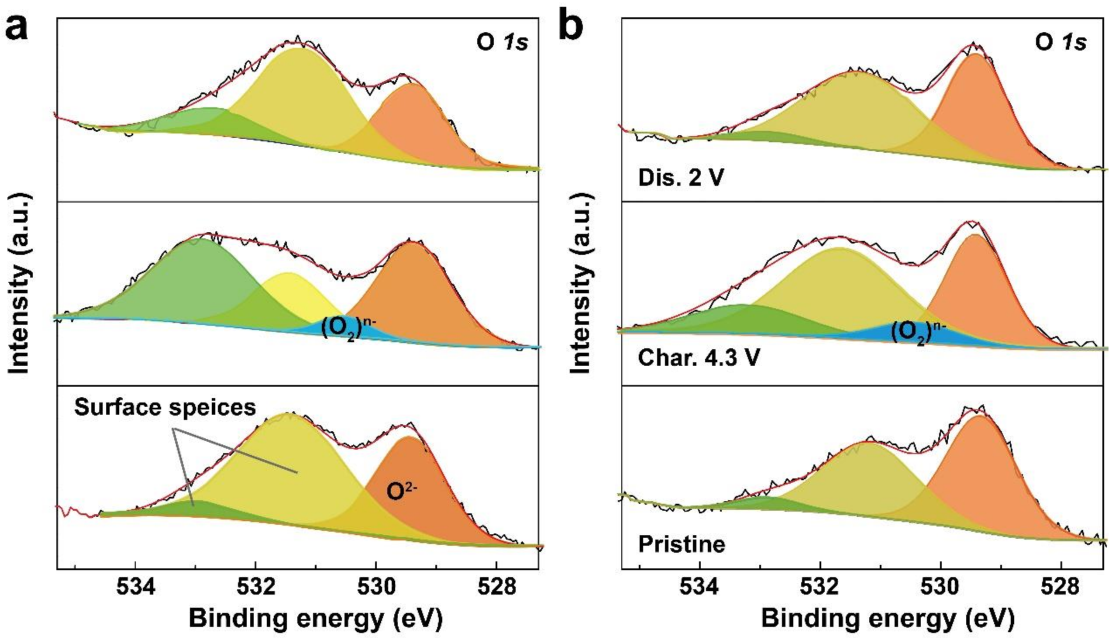

Supplementary Figure 20. *Ex situ* XPS spectra of O*1s* of the NLNFM (**a**) and the NLNFMB (**b**) electrodes at different states in the first cycle.

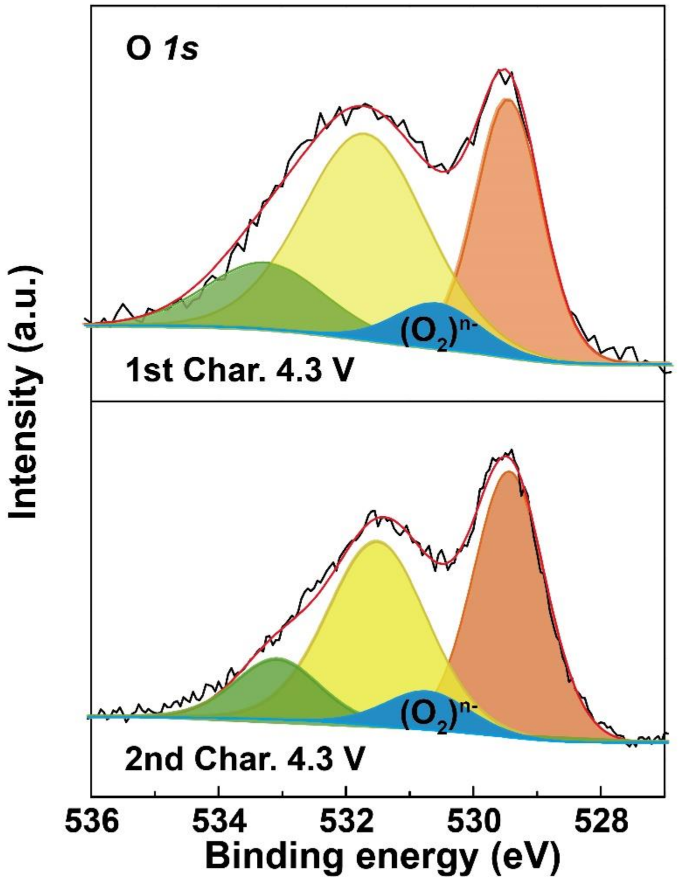

Supplementary Figure 21. *Ex situ* XPS spectra of O*1s* of the NLNFMB electrode at 4.3 V with different cycles.

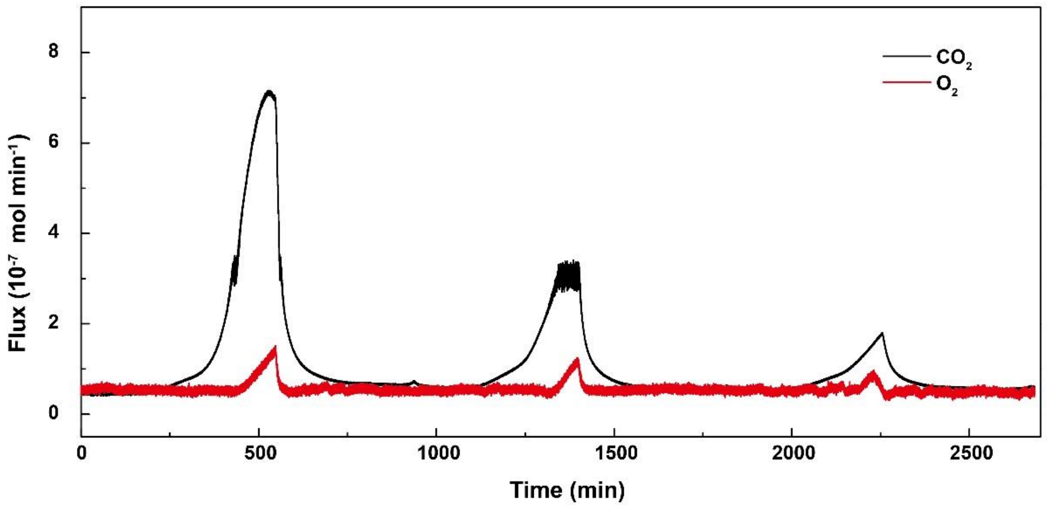

Supplementary Figure 22. DEMS data collected during the initial three cycles of NLNFM electrode.

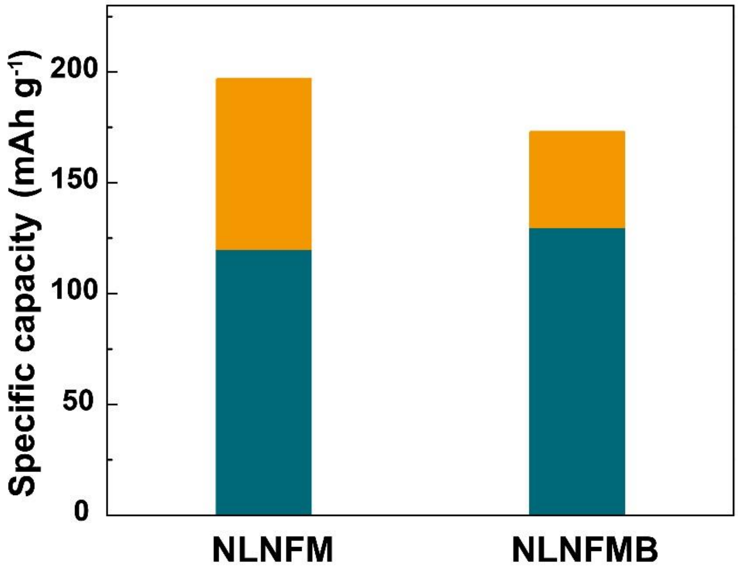

Supplementary Figure 23. Capacity contribution of NLNFM and NLNFMB electrode for the first charging process. The orange part of capacity is contributed by anion redox and the dark cyan part is contributed by cationic redox.

Supplementary Table 1. Summary of dopants for suppressing irreversible oxygen redox reaction.

Dopant	Atomic weight (g mol^{-1})	Bonding energy with oxygen (kJ mol^{-1})	Capacity change ratio (%)	Energy density (Wh kg^{-1})	References
Zn	65.38	≤ 250	-7.3	~480	[1]
Ti	47.87	666.5 ± 5.6	-16	~510	[2]
B	10.81	809	+10.1	521	Our work

Supplementary Table 2. Energy comparison of four structures predicted for NLNFM.

Structure	Energy (eV)
a	-615.53425
b	-616.87002
c	-615.24308
d	-619.65642

We first build a 3×3×1 supercell from the parent crystal [$Na_3Fe_3O_6$] (space group R$\bar{3}$m [166]) and then substitute the three Fe layers by Ni, Mn and Li atoms to obtain [$Na_{27}Mn_{12}Ni_6Fe_6Li_3O_{54}$]. As reported previously, nuclear magnetic resonance spectra, diffraction derived pair-distribution functions, and theoretical calculations all clearly show that TM cations with a valence higher than +3 (average oxidation state of TM in the TMO_2 slab) usually locate adjacent to those with a valence lower than +3 [3-6]. By using this rule, we were able to simplify the screening process. In an ideal lattice configuration, each metal cation shares 1/6th of its charge to each one of the surrounding six oxygen atoms. Therefore, the charge on any oxygen atom is balanced when 1/6th of the total charge contribution from the six neighboring metal cations (which include three TM cations and three Na ions) is +2. In other words, a minimum subunit of the TM layer, that is, a triangular tile of three neighboring TM atoms, is associated with an ideal total charge of +9. In our study, we arranged the TM ordering in our control material according to this rule to predict the lattice structure with lowest energy. In the control compound, Li holds the lowest valence of +1, while Mn has the highest valence of +4. To achieve a total charge of +9, the Li ion in the TM triangle

tends to occupy the neighboring site of Mn. In the TM slabs of $NaLi_{1/9}Ni_{2/9}Fe_{2/9}Mn_{4/9}O_2$, the atomic ratio of Li:Ni:Fe:Mn is 1:2:2:4. Therefore, it is preferrable for one Li atom to be surrounded by four Mn atoms to provide charge balance to the oxygen according to Supplementary Fig.1d. To further verify the lowest energy structure, we arranged other three structures with Li surrounded by less Mn, but more Ni and Fe (a), Fe and Fe (b), Ni and Ni (c), respectively.

Supplementary Table 3. Energy comparison of twelve different B-doping positions in NLNFMB.

B-doping position	Energy (eV)
Li/Mn/Mn (4)	-2505.9751
Li/Fe/Mn (4)	-2506.3587
Ni/Fe/Mn (4)	-2504.3363
Ni/Mn/Mn (4)	-2503.2490
Li/Mn/Mn (3)	-2502.8606
Li/Fe/Mn (3)	-2505.2509
Ni/Fe/Mn (3)	-2503.5966
Ni/Mn/Mn (3)	-2503.2269
Li (3)	-2505.8670
Ni (3)	-2503.9342
Fe (3)	-2505.4533
Mn (3)	-2503.9342

Various substitution strategies have been conducted to find the most energetically favorable configuration with first-principles calculation. Furthermore, to investigate the doping effect of B atom in antiferromagnetic (AFM) configuration, a 2×2×1 supercell is constructed from the previously most stable supercell and 12 different B-doping positions are considered, including tetrahedral interstitial sites surrounded by Li-Mn-Mn (4), Li-Fe-Mn (4), Mn-Fe-Ni (4), Ni-Mn-Mn (4), trigonal sites (three equivalent

apical oxygen atoms of three adjacent MO_6 / NaO_6 octahedrons) surrounded by Li-Mn-Mn (3), Li-Fe-Mn (3), Mn-Fe-Ni (3), Ni-Mn-Mn (3), and triangle plane central sites formed of a MO_6 plane respectively) surrounded by Li (3), Ni (3), Fe (3), Mn (3) respectively. Since there are many trigonal positions, we select the representative sites here to compare the energy. The energy comparison of four different B-doping positions is shown in Supplementary Table 3. Finally, the Bader charge analysis is presented for the lowest-energy B-doping structure, in which B atom is surrounded by Li-Fe-Mn. This identifies the decreased valence of Fe and Mn in the XAS part.

Supplementary Table 4 | Bader charge analysis. Bader charge of the O atoms in the MO_6 Octahedron (M = Ni/Fe/Mn/Li) and in the BO_4 tetrahedron.

O	LiO_6 Bader charge (e)	NiO_6 Bader charge (e)	FeO_6 Bader charge (e)	MnO_6 Bader charge (e)	BO_4 Bader charge (e)
O1	-1.140699	-1.112948	-0.913257	-0.906520	-1.398271
O2	-0.90652	-0.992351	-0.992351	-1.110476	-1.469087
O3	-0.913257	-1.153693	-1.203927	-0.992351	-1.365786
O4	-1.201797	-1.124181	-0.990228	-1.146180	-1.372675
O5	-1.122482	-1.130410	-1.174867	-0.990228	
O6	-0.990228	-1.206591	-1.206591	-1.124181	
Average	-1.045830	-1.120029	-1.080203	-1.044989	-1.401454

Supplementary Table 5 Oxygen vacancy formation energy for NLNFM and NLNFMB.

NLNFM	E(Ovac)/eV	NLNFMB	E(Ovac)/eV
O1	0.17	O1	0.61
O2	0.12	O2	0.41
O3	0.23	O3	0.71
O4	0.24	O4	0.51

Note: O1, O2, O3, O4 are the oxygen atoms in the BO_4 tetrahedron of NLNFMB and the corresponding sites of NLNFM.

Supplementary Table 6. Atomic coordinates and possible occupancies of the unit cell of NLNFM based on Rietveld refinement.

Atom	Mult.	X	Y	Z	Occ.
Na	3a	0.00000	0.00000	0.00000	1
Li	3b	0.00000	0.00000	0.50000	1/9
Ni	3b	0.00000	0.00000	0.50000	2/9
Fe	3b	0.00000	0.00000	0.50000	2/9
Mn	3b	0.00000	0.00000	0.50000	4/9
O	6c	0.00000	0.00000	0.2295	1
a = b= 2.966 Å, c = 16.055 Å R_{wp} = 5.64%, R_p = 4.39%					

Supplementary Table 7. Atomic coordinates and possible occupancies of the unit cell of NLNFMB based on Rietveld refinement.

Atom	Mult.	X	Y	Z	Occ.
Na	3a	0.00000	0.00000	0.00000	1
Li	3b	0.00000	0.00000	0.50000	1/9
Ni	3b	0.00000	0.00000	0.50000	2/9
Fe	3b	0.00000	0.00000	0.50000	2/9
Mn	3b	0.00000	0.00000	0.50000	4/9
O	6c	0.00000	0.00000	0.2259	1
a = b = 2.953Å, c = 16.144 Å R_{wp} = 7.69%, R_p = 5.57%					

Considering the small amount of B, the sites of Na, Li, O and other transition metals will not be changed too much after B-doping. Therefore, we ignore the trace B doping during the crystal structure refinement because it's difficult to affirm the exactly site of B by Rietveld refinement method.

Supplementary Table 8. Slab thickness, d-spacing of Na layer, and interslab distance for NLNFM and NLNFMB.

	NLNFM	NLNFMB
TMO_2 (Å)	2.02	1.91
d-spacing (Å)	3.33	3.47
interslab distance (Å)	5.35	5.38

Supplementary Table 9. Electrochemical performance comparison between the hard carbon || NLNFMB full cell and other Na-ion full cells reported.

Full cell	Specific energy (Wh/kg)	Cell configuration	Active material in positive electrode (wt%)	Electrolyte	Cut-off voltage window (V)	Specific current (mA /g)	Cycle number (n)	Ref.
Hard carbon || $Na_{7/9}Cu_{2/9}Fe_{1/9}Mn_{2/3}O_2$	195	Coin cell	80	0.8 M $NaClO_4$ in EC:DEC (1:1)	1–4.2	20	50	7
C || $Na_1(Fe_{0.5}Mn_{0.5})O_2$ + 10%Na_3P	210	Coin cell	80	1M $NaClO_4$ in EC:DMC (1:1)	0-4.3	13	20	8
Hard carbon || P2/P3-$Na_{0.7}Li_{0.06}Mg_{0.06}Ni_{0.22}Mn_{0.67}O_2$	218	Pouch cell	75	1M $NaClO_4$ in PC (5%FEC)	2.0–4.2	12	50	9
Hard carbon || NLNFMB	224	Coin cell	80	1M $NaPF_6$ in EC:DEC (1:1, 5%FEC)	1.5-4.2	250	100	Our work

Supplementary References

1. Zhang, K. *et al.* Manganese based layered oxides with modulated electronic and thermodynamic properties for sodium ion batteries. *Nat. Commun.* **10,** 5203 (2019).

2. Li, C. *et al.* Unraveling the critical role of Ti substitution in P2-$Na_xLi_yMn_{1-y}O_2$ cathodes for highly reversible oxygen redox chemistry. *Chem. Mater.* **32,** 1054-1063 (2020).

3. Van der Ven, A. & Ceder, G. Ordering in Lix($Ni_{0.5}Mn_{0.5}$)O_2 and its relation to charge capacity and electrochemical behavior in rechargeable lithium batteries. *Electrochem. Commun.* **6,** 1045-1050 (2004).

4. Dixit, M. *et al.* Thermodynamic and kinetic studies of $LiNi_{0.5}Co_{0.2}Mn_{0.3}O_2$ as a positive electrode material for Li-ion batteries using first principles. *Phys. Chem. Chem. Phys.* **18,** 6799-6812 (2016).

5. Harris, K. J. *et al.* Structure solution of metal-oxide Li battery cathodes from simulated annealing and lithium NMR spectroscopy. *Chem. Mater.* **29,** 5550-5557 (2017).

6. Kunnikuruvan, S., Chakraborty, A. & Major, D. T. Monte carlo- and simulated-annealing-based funneled approach for the prediction of cation ordering in mixed transition-metal oxide materials. *J. Phys. Chem. C* **124,** 27366-27377 (2020).

7. Li, Y. *et al.* Air-stable copper-based P2-$Na_{7/9}Cu_{2/9}Fe_{1/9}Mn_{2/3}O_2$ as a new positive electrode material for sodium-ion batteries. *Adv. Sci.* **2,** 1500031 (2015).

8. Zhang, B. et al. Insertion compounds and composites made by ball milling for advanced sodium-ion batteries. *Nat. Commun.* **7,** 10308 (2016).

9. Zhou, Y.-N. *et al.* A P2/P3 composite layered cathode for high-performance Na-ion full batteries. *Nano Energy* **55,** 143-150 (2019).
